# PEXEL is a proteolytic maturation site for both exported and non-exported *Plasmodium* proteins

**DOI:** 10.1128/msphere.00393-23

**Published:** 2024-02-09

**Authors:** Manuel A. Fierro, Ajla Muheljic, Jihui Sha, James Wohlschlegel, Josh R. Beck

**Affiliations:** 1 Department of Biomedical Sciences, Iowa State University, Ames, lowa, USA; 2 Roy J. Carver Department of Biochemistry, Biophysics and Molecular Biology, Iowa State University, Ames, Iowa, USA; 3 Department of Biological Chemistry, University of California, Los Angeles, California, USA; Australian National University, Canberra, Australia

**Keywords:** PEXEL, Plasmepsin V, *Plasmodium*, export, UIS2, RON11, Plasmepsin IX

## Abstract

Obligate intracellular malaria parasites dramatically remodel their erythrocyte host through effector protein export to create a niche for survival. Most exported proteins contain a pentameric *Plasmodium* export element (PEXEL)/host-targeting motif that is cleaved in the parasite ER by the aspartic protease Plasmepsin V (PMV). This processing event exposes a mature N terminus required for translocation into the host cell and is not known to occur in non-exported proteins. Here, we report that the non-exported parasitophorous vacuole protein UIS2 contains a *bona fide* PEXEL motif that is processed in the *P. falciparum* blood stage. While the N termini of exported proteins containing the PEXEL and immediately downstream ~10 residues are sufficient to mediate translocation into the RBC, the equivalent UIS2 N terminus does not promote the export of a reporter. Curiously, the UIS2 PEXEL contains an unusual aspartic acid at the fourth position, which constitutes the extreme N-terminal residue following PEXEL cleavage (P1′, RIL↓DE). Using a series of chimeric reporter fusions, we show that Asp at P1′ is permissive for PMV processing but abrogates export. Moreover, mutation of this single UIS2 residue to alanine enables export, reinforcing that the mature N terminus mediates export, not PEXEL processing *per se*. Prompted by this observation, we further show that PEXEL sequences in the N termini of other non-exported rhoptry proteins are also processed, suggesting that PMV may be a more general secretory maturase than previously appreciated, similar to orthologs in related apicomplexans. Our findings provide new insight into the unique N-terminal constraints that mark proteins for export.

**IMPORTANCE:**

Host erythrocyte remodeling by malaria parasite-exported effector proteins is critical to parasite survival and disease pathogenesis. In the deadliest malaria parasite *Plasmodium falciparum*, most exported proteins undergo proteolytic maturation via recognition of the pentameric *Plasmodium* export element (PEXEL)/host-targeting motif by the aspartic protease Plasmepsin V, which exposes a mature N terminus that is conducive for export into the erythrocyte host cell. While PEXEL processing is considered a unique mark of exported proteins, we demonstrate that PEXEL motifs are present and processed in non-exported proteins. Importantly, we show that specific residues at the variable fourth position of the PEXEL motif inhibit export despite being permissive for processing, reinforcing that features of the mature N terminus, and not PEXEL cleavage, identify cargo for export. This opens the door to further inquiry into the nature and evolution of the PEXEL motif.

## INTRODUCTION

*Plasmodium* spp. are highly dependent on protein export for survival within the red blood cell (RBC) host. These proteins remodel the RBC to facilitate nutrient acquisition by the parasite and evasion of vertebrate host defenses (1). Proteins destined for export enter the secretory pathway via a signal sequence or transmembrane domain and are deposited into a vacuolar niche formed during invasion called the parasitophorous vacuole (PV) (2). Once in the PV, exported proteins are unfolded and transported across the PV membrane (PVM) via the PTEX complex to reach their final destination in the RBC space (3–7).

In *Plasmodium falciparum*, most exported proteins contain a *Plasmodium* export element (PEXEL, also known as the host-targeting signal), a pentameric motif (RxLxE/Q/D) generally located downstream of a recessed signal sequence (8, 9). Entry into the endoplasmic reticulum (ER) occurs through a distinct SEC translocon complex that includes the SEC61 channel and SPC25, a non-catalytic component of the signal peptidase complex (10). During entry, the PEXEL sequence is recognized and cleaved between the third and fourth residues by Plasmepsin V (PMV), an aspartic protease that appears to operate in place of the classical signal peptidase SPC21 (11, 12). In keeping with ER translocation through a distinct SEC61 complex, the signal sequence of PEXEL proteins is generally not efficiently cleaved by signal peptidase and appears to act as a stable signal anchor that is removed through the action of PMV (13, 14). Following PMV cleavage, the newly exposed mature N terminus is acetylated (15), although this widespread modification is not specific to exported proteins (16, 17) and is not sufficient for export (18).

PEXEL processing is crucial as export is blocked in non-cleaved mutants, which accumulate in the ER or PV (12, 18–22). Importantly, however, PMV processing can be bypassed by a reporter engineered to be processed by an exogenous protease to generate the equivalent of a mature PEXEL N terminus (21, 23). This indicates that PMV is not directly involved in cargo transfer in the export pathway but rather exposes an export-mediating mature N terminus that is recognized by PTEX. While the mature protein retains only the last two PEXEL residues (xE/Q/D), the ~10 residues immediately downstream of the PEXEL are also important for export but do not contain a discernable motif, suggesting that the N-terminal secondary structure adopted following processing may be important (19–21, 24, 25). Furthermore, while PEXEL processing is necessary for the export of proteins that contain this motif, it is not strictly required for translocation into the host cell as several PEXEL-negative exported proteins (PNEPs) are known, which also require PTEX translocation across the PVM but are not substrates for PMV (5, 6, 26). Indeed, despite the lack of PMV processing, the mature PNEP N terminus is functionally equivalent to the mature PEXEL N terminus in mediating export, indicating that PEXEL processing is not strictly necessary for this process (19).

Although processing and export have not been validated for the full repertoire of PEXEL-containing proteins (20, 27–29), PEXEL cleavage is highly predictive for export and thought to be constrained to proteins that are trafficked into the erythrocyte. Interestingly, PEXEL processing has also been observed during the liver stage in proteins that are not exported beyond the PV into the hepatocyte (30, 31). As several of these proteins are also expressed in the blood stage and in some cases are translocated into the erythrocyte, this may correspond to a mechanistic difference in export between the blood and liver stages; however, for the purple acid phosphatase domain-containing protein UIS2, localization also appears to be constrained to the PV during the blood stages (32, 33), suggesting a class of non-exported proteins exists that contain *bona fide* PEXEL motifs.

Here, we show that although the UIS2 PEXEL is processed and N-acetylated in blood-stage *P. falciparum,* the mature protein is not translocated beyond the PV, nor is the UIS2 leader sequence able to promote the export of a fluorescent reporter despite PEXEL cleavage. Remarkably, a single amino acid change in the fourth position of the UIS2 PEXEL from aspartic acid to alanine enabled the export of this reporter, showing that this variable residue, which constitutes the first N-terminal amino acid after processing (P1′), is important for mediating export. Prompted by our findings with UIS2, we further identified PEXEL cleavage in several non-exported rhoptry proteins, some of which also contain a P1′ residue that restricts export. Collectively, our results indicate that PEXEL processing is a more general proteolytic maturation event than previously appreciated and reinforce that the mature N terminus and not PEXEL processing *per se* identifies cargo for export. This work provides new insight into the unique N-terminal constraints that mark proteins for export and opens the door for further analysis of the nature and function of the PEXEL motif outside the context of exported effectors.

## RESULTS

### The PV protein UIS2 harbors a *bona fide* PEXEL motif that is not permissive for export

**Fig 1:**
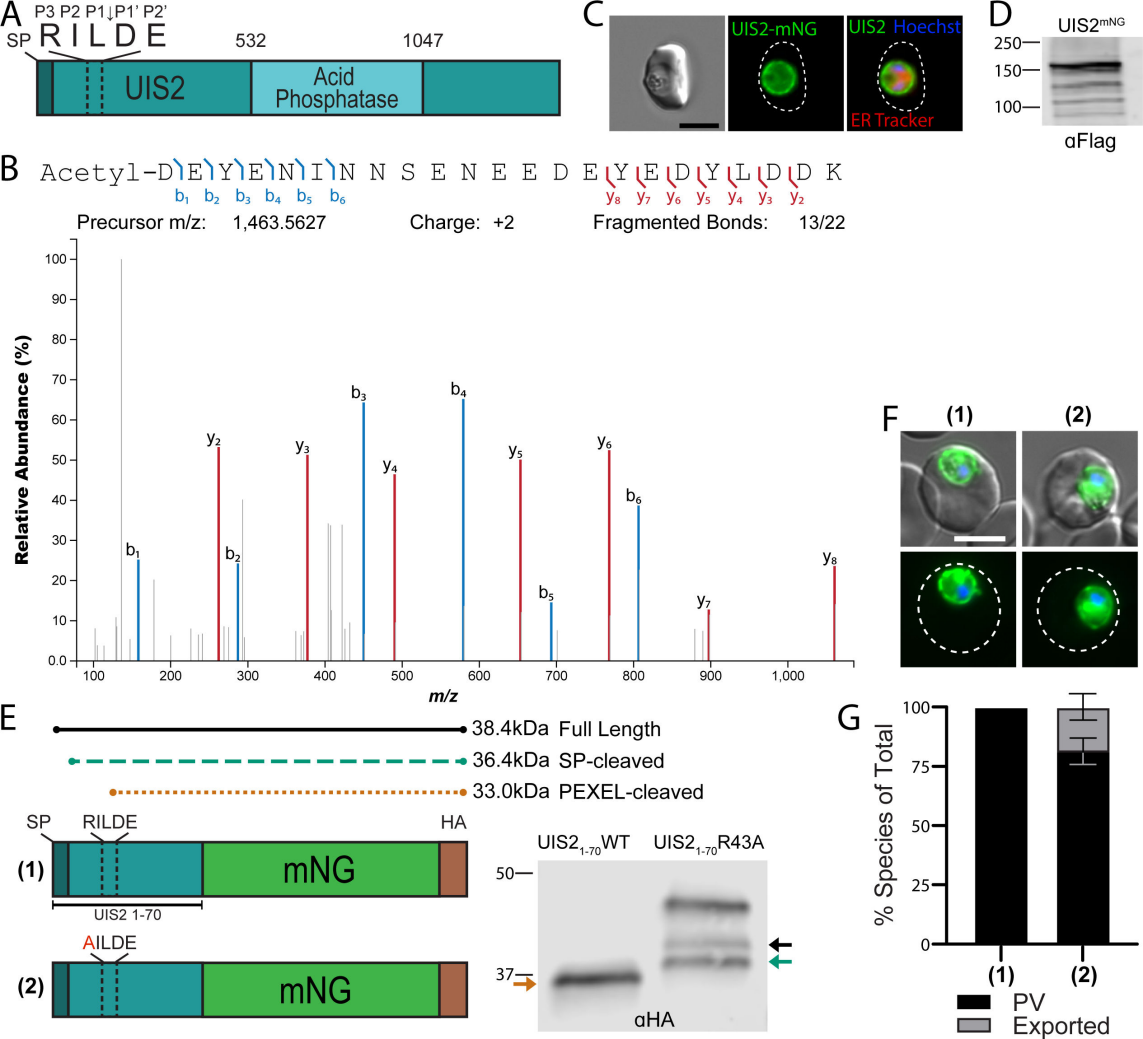
The PEXEL of UIS2 is cleaved but does not result in export. (**A**) Schematic of full-length UIS2. Dark teal bar indicates signal peptide; dotted box indicates the location of the PEXEL motif. (**B**) Mass spectra of the N-terminal most UIS2 peptide detected by proximity labeling with EXP2-BioID2. The spectra correspond to PEXEL processing after L45 and acetylation at the new N terminus. (**C**) Representative live microscopy of parasites where UIS2 is endogenously tagged with mNeonGreen (mNG)-3xFlag. Cells were stained with ER tracker and Hoechst 33342. Dashed line indicates RBC boundary traced from Differential Interference Contrast (DIC). (**D**) Western blot of UIS2-mNG-3xFlag parasites using anti-Flag antibodies. The expected size after PEXEL cleavage is 196 kDa. (**E**) Schematic of the reporter construct containing residues 1–70 of UIS2 with either the WT PEXEL motif or an R43A mutation fused to mNG and expressed under the control of the *cam* promoter. Western blot of reporter constructs showing size differences indicating the presence or absence of PEXEL cleavage. The expected molecular weights are labeled above the reporter schematic. The corresponding colored arrows are shown on the blot image to denote the full-length, signal peptidase-cleaved or PEXEL-cleaved versions of the reporters. (**F**) Representative live microscopy of parasites expressing each reporter from panel **E**. Bottom images show mNG (green) and Hoechst 33342 (blue) fluorescence and top images show merge with DIC. Dashed line indicates RBC boundary traced from DIC. (**G**) Quantification of reporter localization as exported into the RBC or strictly within the PV/parasite. Data are presented as means ± standard deviation from two biological replicates [(1) *n* = 49 iRBCs and (2) *n* = 65 iRBCs]. For statistical analysis of reporter export, see Fig. S2B. All scale bars: 5 µm.

To query whether PEXEL processing extends to proteins that are not translocated into the RBC during intraerythrocytic development, we examined UIS2, a phosphatase domain-containing PV protein that encodes a classical signal peptide (residues 1–21) followed by a PEXEL motif (RILDE, residues 43–47) (Fig. 1A) (32–34). Intriguingly, PEXEL processing of the *Plasmodium berghei* ortholog of UIS2 (RVL↓QE) was detected by mass spectrometry analysis of liver-stage parasites (30) but has not been evaluated in the blood stage. To determine if the *P. falciparum* UIS2 PEXEL is also processed during the blood stage, we searched a previous mass spectrometry data set that was generated from proximity labeling with a BioID2 fusion to the PV protein EXP2 in which UIS2 was well represented (Fig. S1) (35). Indeed, peptide spectra corresponding to the processed, N-acetylated UIS2 PEXEL (cleavage after L45, Acetyl-DEYENINNSENEEDEYEDYLDDK) were identified, indicating similar PEXEL processing of *P. falciparum* UIS2 in the blood stage (Fig. 1B). To validate that UIS2 is not exported into the RBC, we generated a C-terminal mNeonGreen (mNG) fusion to the endogenous copy of UIS2, which trafficked to the parasite periphery but was not observed in the erythrocyte (Fig. 1C and D), consistent with strict localization to the PV as previously reported (32, 33). To ensure UIS2 was not translocated into the host cell and associated to the cytoplasmic face of the PVM, we performed serial permeabilization of the RBC membrane and PVM using Streptolysin-O and saponin, respectively, followed by treatment with Proteinase K to degrade accessible mNG. Importantly, proteinase treatment did not alter mNG fluorescence in the Streptolysin O-treated cells but drastically reduced fluorescence in the saponin-treated cells similar to the non-exported PV resident protein PTEX150, confirming that UIS2 is contained within the PVM and not exported (Fig. S2A).

The N termini of PEXEL-containing exported proteins, including the PEXEL and immediately downstream spacer region (~10 residues), are able to mediate export when fused to a fluorescent reporter protein (8, 9, 18, 36). To determine if more C-terminal features of UIS2 prevent its export, we fused the first 70 amino acids of UIS2, including the PEXEL and downstream 23 residues, to mNG with a C-terminal 3xHA epitope tag (Fig. 1E). This cassette was placed under the control of the *cam* promoter and the plasmid was stably inserted into the *attB* site on chromosome 6 in NF54^attB^ parasites. Western blot analysis showed a single band at the expected size for PEXEL cleavage (Fig. 1E, first lane), and live fluorescence indicated that this fusion protein was also secreted to the PV but failed to be exported into the erythrocyte (Fig. 1F and G). To ensure PEXEL processing was still occurring in the context of the fusion protein, we generated a version where the PEXEL P3 arginine was changed to an alanine (R43A), which prevents processing by PMV (20). Consistent with previous reports (12, 18, 20), this mutation produced an upshift in molecular weight to the expected size for the signal peptidase-cleaved version of the protein as well as a minor band that likely represents the full-length, unprocessed form (Fig. 1E, second lane, green and black arrows, respectively). An additional band that migrated at a higher molecular weight than the predicted size of the full-length protein was also observed in the mutated reporter, possibly indicating post-translational modification of residues N-terminal to the PEXEL motif. Localization of the R43A mutant was indistinguishable from the PEXEL-processed fusion, trafficking to the PV but not the host cell (Fig. 1F and G; Fig. S2B). Collectively, these results show that UIS2 contains a *bona fide* PEXEL motif, but processing does not yield a mature N terminus that can promote export, in contrast with other PEXEL-containing exported proteins.

### UIS2 PEXEL position P1′ is not conducive for export

**Fig 2:**
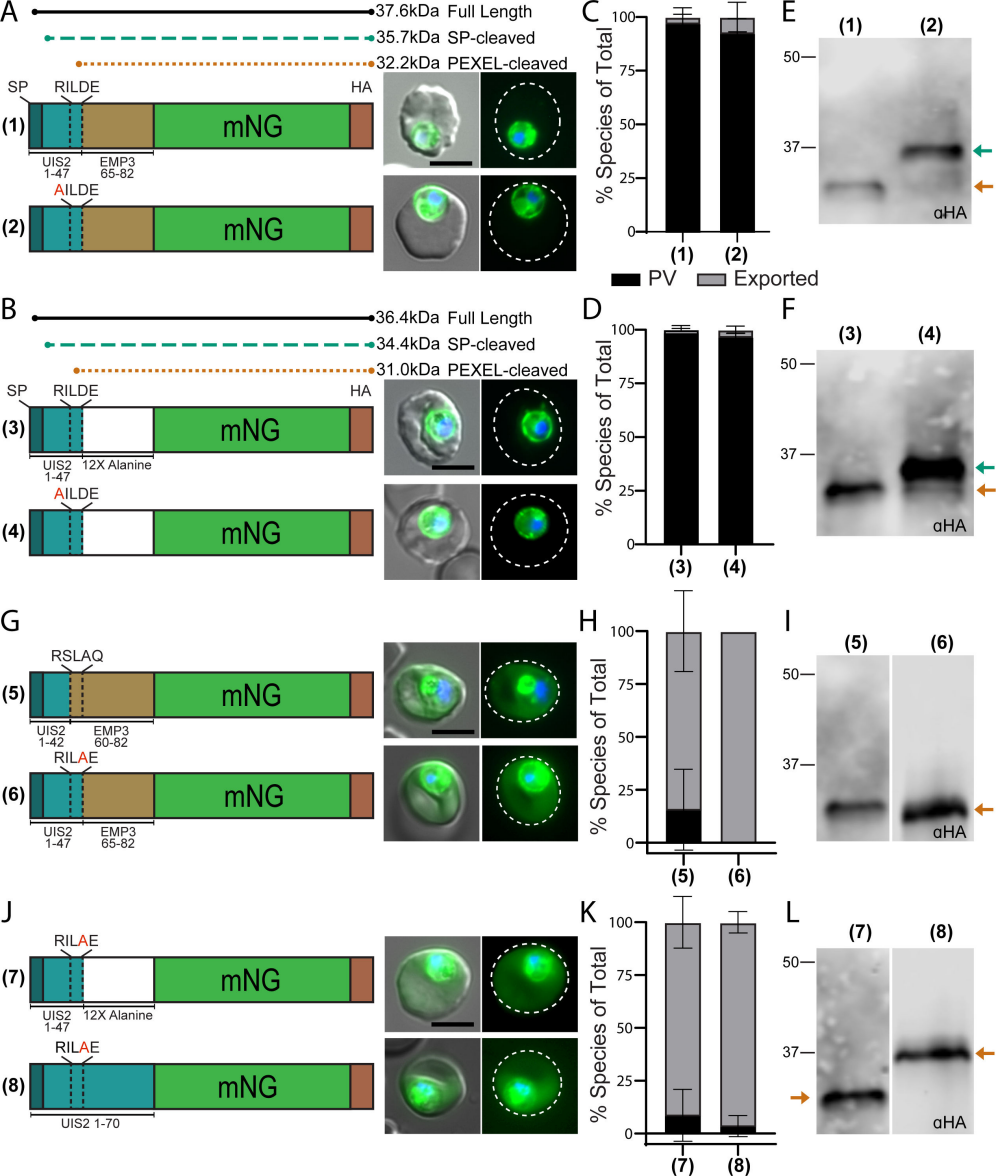
Aspartic acid at P1′ in the UIS2 PEXEL is not permissive for export. (**A**) Schematic and representative live microscopy images of UIS2-EMP3 chimeric constructs containing residues 1–47 of UIS2 fused to residues 65–82 of EMP3 with either the wild-type UIS2 PEXEL (1) or containing an R43A mutation of the UIS2 PEXEL (2). (**B**) Schematic and representative live microscopy images of UIS2-A12 chimeric constructs containing residues 1–47 of UIS2 fused to 12 alanine residues with the wild-type UIS2 PEXEL (3) or an R43A mutation (4). (**C and D**) Quantification of reporter localization as exported into the RBC or strictly within the PV/parasite. Data are presented as means ± standard deviation from two biological replicates [(1) *n* = 79 iRBCs, (2) *n* = 76 iRBCs, (3) *n* = 77 iRBCs, and (4) *n* = 67 iRBCs]. (**E and F**) Western blot of chimeric constructs from panels **A** and **B**. Arrows indicate bands corresponding to the expected molecular weights shown in the schematic in panel **A** or **B** for signal peptidase-cleaved or PEXEL-cleaved versions of the reporters. (**G**) Schematic and representative live microscopy images of UIS2-EMP3 chimeric constructs containing residues 1–42 of UIS2 fused to residues 60–82 of EMP3 (5, EMP3 PEXEL) or residues 1–47 of UIS2 fused to residues 65–82 of EMP3 with a R46A mutation (6, UIS2 PEXEL R46A). (**H**) Quantification of reporter localization as exported into the RBC or strictly within the PV/parasite. Data are presented as means ± standard deviation from two biological replicates [(5) *n* = 72 iRBCs and (6) *n* = 53 iRBCs]. (**I**) Western blot of chimeric constructs from panel **G**. **(J**) Schematic and representative live microscopy images of constructs containing residues 1–47 of UIS2 fused to 12 alanine residues with a D46A mutation (7) or residues 1–70 of UIS2 with an D46A mutation (8). (**K**) Quantification of reporter localization as exported into the RBC or strictly within the PV/parasite. Data are presented as means ± standard deviation from two biological replicates [(7) *n* = 60 iRBCs and (8) *n* = 139 iRBCs]. (**L**) Western blot of chimeric constructs from panel **J**. All scale bars: 5 µm. Microscopy images show mNG (green) and Hoechst 33342 (blue) fluorescence on the right and merge with DIC on the left. Dashed lines indicate the RBC boundary traced from DIC. For statistical analysis of reporter export, see Fig. S2B through D.

The ~10–20 residues exposed upon PEXEL processing are critical for export, suggesting that the mature N terminus of UIS2 lacks the necessary information to be recognized as cargo by PTEX. To determine if UIS2 residues immediately downstream of the PEXEL prevent export, we generated a mNG fusion reporter that replaced UIS2 residues 48–70 with residues 65–82 of the exported protein EMP3 previously shown to be sufficient for export in combination with the EMP3 PEXEL (Fig. 2A) (20). In parallel, we placed a stretch of 12 alanine residues after the UIS2 PEXEL as this is also capable of mediating export when positioned immediately downstream of an export-competent PEXEL (Fig. 2B) (20). In both cases, we surprisingly observed no export of the reporter into the RBC (Fig. 2A through D; Fig. S2C and D). To ensure this was not the result of an uncleaved PEXEL, we generated R43A mutant versions of these constructs and observed the expected upshift in molecular weight consistent with abrogation of PEXEL processing, confirming that the lack of export was not attributable to impaired UIS2 PEXEL processing in these chimeric constructs (Fig. 2E and F).

Lack of export in these chimeras indicates that either residues upstream of the UIS2 PEXEL or the PEXEL itself are inhibitory for export. To differentiate between these two possibilities, we replaced the UIS2 PEXEL with the PEXEL of EMP3 (RILDE > RSLAQ) in the UIS2-EMP3 chimera and found that this enabled export of mNG (Fig. 2G through I; Fig. S2C). These results demonstrate that residues within the UIS2 PEXEL motif prohibit export, presumably amino acids at P1′ or P2′ that remain at the mature N terminus. Previous reports have shown that mutations at the P1′ position of the PEXEL can inhibit the export of chimeric reporters with a minor impact on PEXEL processing (19, 21, 28). While serine, alanine, cysteine, threonine, and tyrosine are common at the P1′ position, the UIS2 PEXEL contains an unusual aspartic acid at P1′, which is not found in other predicted or identified exported proteins, suggesting this residue may not be compatible with an export-competent mature N terminus (20, 28, 29). Indeed, replacing this aspartic acid with an alanine in both the UIS2_1-47_-EMP3_65-82_ (Fig. 2G through I; Fig. S2C) and UIS2_1-47_-12xAlanine chimeras or the original UIS2 reporter (Fig. 2J through L; Fig. S2B and D) enabled export. As expected, the D > A mutation at P1′ did not impact the PEXEL processing of these constructs (Fig. 2I and L). These results show that aspartic acid at P1′ is permissive for processing but not for export, reinforcing that the mature N terminus, and not PEXEL processing *per se,* identifies cargo for export.

### Cleavable PEXEL motifs are present in other secreted proteins that do not traffic to the PV

**Fig 3:**
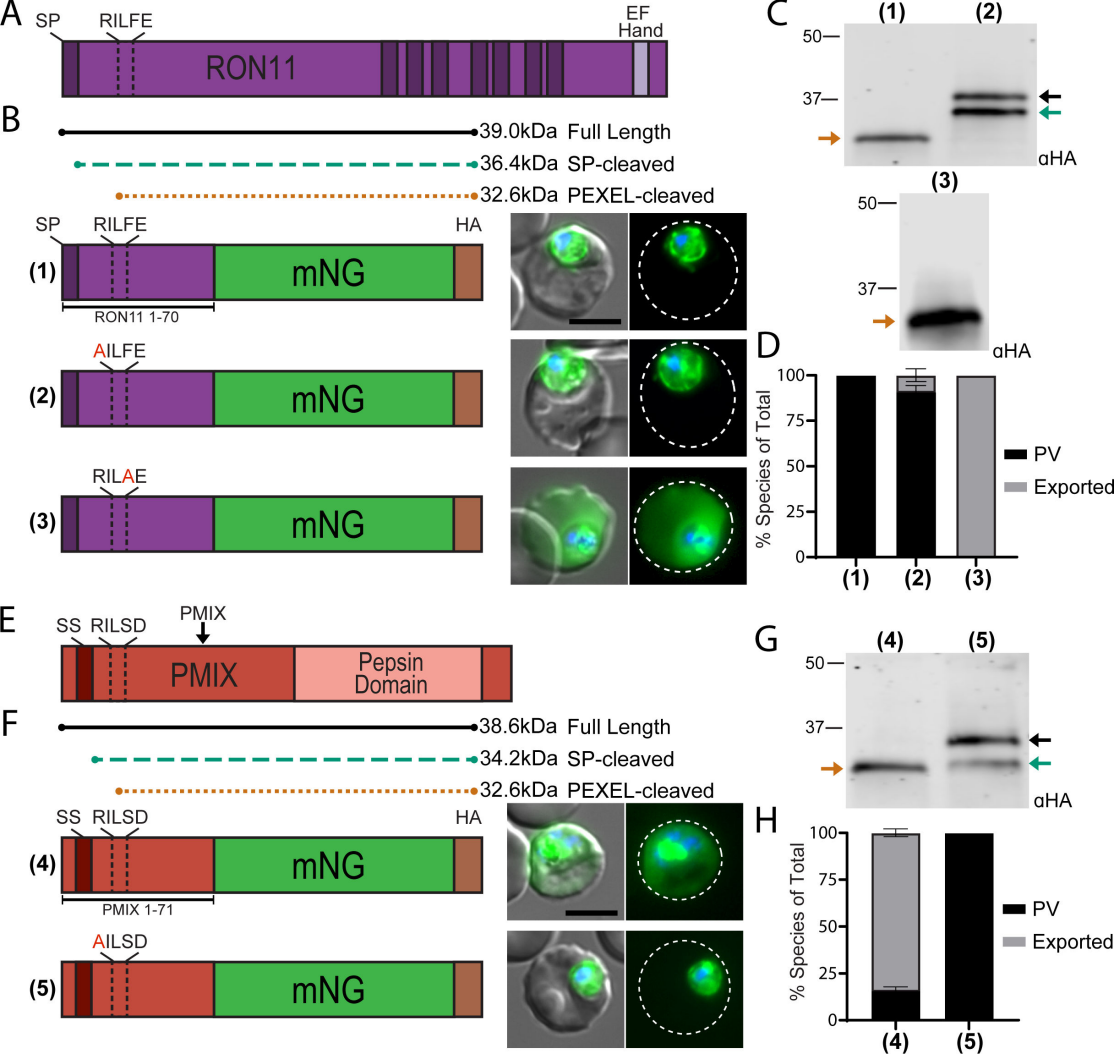
PEXEL motifs are present and cleaved in the N termini of the rhoptry proteins RON11 and PMIX. (**A**) Schematic of full-length RON11. Dark bars represent either the signal peptide or transmembrane domains. (**B**) Schematic of the reporter construct containing residues 1–70 of RON11 with either the WT PEXEL motif (1), an R51A (2), or F54A (3) PEXEL mutation, followed by representative live microscopy of parasites expressing the reporter. (**C**) Western blot of RON11 reporter constructs. Arrows indicate bands corresponding to the expected molecular weights shown in the schematic in panel **B** for the full-length, signal peptidase-cleaved or PEXEL-cleaved versions of the reporters. (**D**) Quantification of reporter localization as exported into the RBC or strictly within the PV/parasite. Data are presented as means ± standard deviation from two biological replicates [(1) *n* = 74 iRBCs, (2) *n* = 135 iRBCs, and (3) *n* = 53 iRBCs]. (**E**) Schematic of full-length PMIX. Dark red bar represents the recessed signal sequence. (**F**) Schematic of the reporter construct containing residues 1–71 of PMIX with either the WT PEXEL motif (4) or an R46A mutation (5) followed by representative live microscopy of parasites expressing the reporter. (**G**) Western blot of PMIX reporter constructs. Arrows indicate bands corresponding to the expected molecular weights shown in the schematic in panel **F** for the full-length, signal peptidase-cleaved or PEXEL-cleaved versions of the reporters. (**H**) Quantification of reporter localization as exported into the RBC or strictly within the PV/parasite. Data are presented as means ± standard deviation from two biological replicates [(4) *n* = 99 iRBCs and (5) *n* = 92 iRBCs]. All scale bars: 5 µm. Microscopy images show mNG (green) and Hoechst 33342 (blue) fluorescence on the right and merge with DIC on the left. Dashed lines indicate the RBC boundary traced from DIC. For statistical analysis of reporter export, see Fig. S2E and F.

Prompted by our observations with UIS2, we explored whether cleavable PEXELs were also present in other non-exported proteins. Prediction analyses have identified PEXEL motifs in rhoptry proteins, which enter the secretory pathway but are not trafficked to the PV nor exported into the host cell (20, 27–29). As such, we focused on the rhoptry-localized proteins RON11 and Plasmepsin IX (PMIX) (37–39). RON11 is a large protein containing a classical signal peptide (residues 1–22) followed by an N-terminal PEXEL motif (RILFE, residues 51–55). This protein also contains seven transmembrane domains and a single C-terminal EF-Hand motif (Fig. 3A). Intriguingly, previous studies showed that the RON11 N terminus containing the PEXEL was not conducive to the export of a GFP reporter, but export was enabled via an F54A mutation of the putative P1′ residue (27, 28). However, the PEXEL processing of these RON11 reporters was not evaluated. To determine if the RON11 PEXEL is cleaved, we fused wild-type (WT) and R51A versions of residues 1–70 of RON11 to the mNG-3xHA reporter (Fig. 3B). The WT RON11 construct yielded a single band at the expected size for PEXEL processing, while the R51A mutant produced upshifted bands with molecular weights corresponding to full-length and signal peptidase-cleaved species, indicating the RON11 PEXEL is cleaved (Fig. 3C). Regardless of PEXEL processing, we observed a strict PV localization of both the control and R51A RON11 reporters with no evidence of export beyond the PV (Fig. 3B and D; Fig. S2E). Similar to UIS2, mutating the RON11 P1′ residue to alanine (F54A, RILFE > RILAE) enabled export of mNG, demonstrating that phenylalanine at P1′ is compatible with PEXEL cleavage but also prohibits export (Fig. 3B through D; Fig. S2E).

We next examined the rhoptry protease PMIX, which contains an N-terminal hydrophobic sequence that may function as a classical signal peptide or recessed signal sequence (residues 1–39) followed by an N-terminal PEXEL (RILSD, residues 46–50) in a pro-region upstream of the aspartic peptidase domain (Fig. 3E). Fusion of residues 1–71 of PMIX to an mNG reporter produced a single band at the expected size for the PEXEL cleaved species, while an R46A mutation resulted in upshifted bands with molecular weights corresponding to full-length and signal peptidase-cleaved species, showing the PMIX PEXEL is processed in the blood stage (Fig. 3F and G). Unlike the unusual aspartic acid or phenylalanine P1′ residues found in UIS2 or RON11, the PMIX PEXEL encodes a P1′ serine, which is common among exported PEXEL proteins. Remarkably, the PMIX reporter fusion was exported, consistent with the importance of P1′ for determining the export competence of the mature PEXEL N terminus (Fig. 3F and H; Fig. S2F). As expected, the R46A mutation blocked PEXEL processing and eliminated export (Fig. 3F through H; Fig. S2F). Taken together, these results show that PEXEL processing occurs not only in non-exported resident PV proteins but also in proteins that traffic to the rhoptries.

### Recessed signal sequence positioning in exported proteins is important for PEXEL processing

Many PEXEL-containing exported proteins are structured with an N-terminal hydrophobic sequence (sometimes referred to as “recessed signal sequence”) within a short first exon followed by the PEXEL motif encoded near the beginning of the second exon (8, 27). The recessed positioning of the signal sequence in exported proteins is thought to serve as a signal anchor in a type II membrane protein configuration and may be important for ER entry through a distinct SEC61 translocon complex that contains PMV in place of signal peptidase (10). In contrast, RON11 and UIS2 possess a canonical signal peptide positioned at their extreme N terminus, which is expected to mediate ER entry and signal peptide processing through the classical SEC61 complex associated with the signal peptidase complex (Fig. 1A and 3A) (10). The classical signal peptide configuration in UIS2 and RON11 suggests that the “recessed” signal sequence positioning in exported proteins may not be important for PEXEL processing and export. Indeed, replacing the EMP3 sequence upstream of the PEXEL (which contains a recessed signal sequence) with the equivalent region from UIS2 (which contains a signal peptide at the extreme N terminus) supports the export of mNG (Fig. 2G), similar to previous observations indicating a recessed signal sequence is not necessary for PEXEL processing and export (20). Moreover, many members of the expanded RIFIN and STEVOR families of exported proteins are predicted to contain a signal peptide, further suggesting that multiple modes of ER entry can accommodate PEXEL processing.

**Fig 4:**
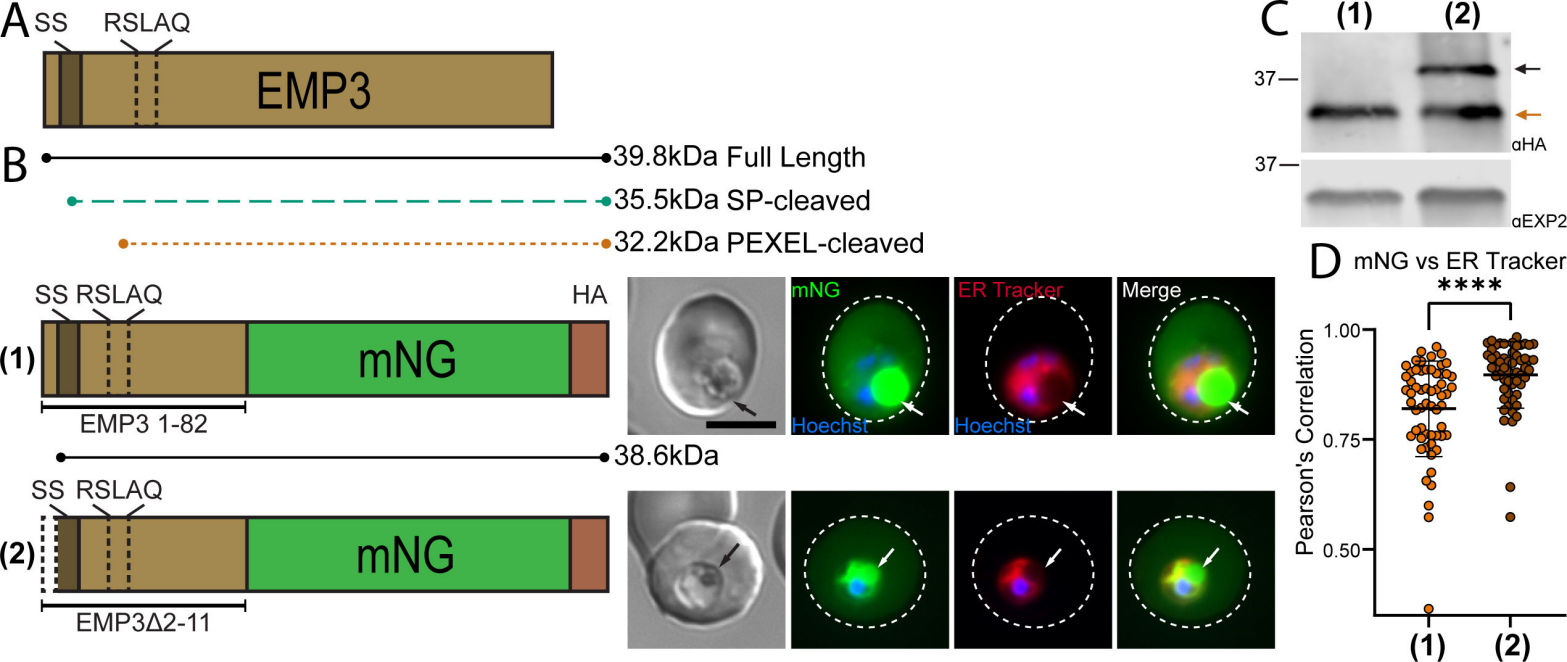
The position of the recessed signal sequence is important for PEXEL cleavage in EMP3. (**A**) Schematic of full-length EMP3. Dark brown bar represents the recessed signal sequence. (**B**) Schematic and representative live microscopy images of reporter constructs containing the WT recessed signal sequence or N-terminal truncation of residues 2–11 to place the signal sequence immediately after the start methionine. The mNG signal within the parasite in the WT reporter line corresponds to the digestive vacuole (visible in the DIC panel and indicated by arrows), typical for proteins secreted to the PV or exported into the host cell and endocytosed along with host cytosol. The mNG signal within the parasite in the truncated reporter line is also within the digestive vacuole but additionally perinuclear. Dashed lines indicate RBC boundary traced from DIC. Scale bar: 5 µm. For statistical analysis of reporter export, see Fig. S2G. (**C**) Western blot of WT and truncated EMP3 constructs. Arrows indicate bands corresponding to the expected molecular weights shown in the schematic in panel **B** for full-length and PEXEL-cleaved forms. Staining for EXP2 was used as a loading control. (**D**) Quantification of Pearson’s correlation coefficient between mNG and ER tracker using Cell Profiler (41). Data are presented as means ± standard deviation from two biological replicates [(1) *n* = 59 iRBCs, (5) *n* = 57 iRBCs; ****P < 0.0001; unpaired *t* test].

Since classical signal peptide and recessed signal sequence N termini appear to be fully interchangeable for PEXEL processing, we tested whether a recessed signal sequence can function as a classical signal peptide when positioned at the extreme N terminus. For this, we fused EMP3 residues 1–82 to mNG and compared this WT export reporter to a version where we removed residues 2–11 to place the hydrophobic stretch immediately after the start codon, mimicking the positioning of a classical signal peptide (Fig. 4A and B). SignalP 3 (40) predicts a signal peptide for this arrangement with cleavage 24 residues upstream of the PEXEL, similar to the predicted signal peptidase cleavage sites for UIS2 and RON11, which are 26 and 28 residues upstream of the PEXEL, respectively (Fig. S3). The truncated reporter was still exported at comparable levels to the WT control (Fig. 4B; Fig. S2G). However, western blot showed inefficient processing of the truncated reporter, suggesting that the hydrophobic region of EMP3 is not efficiently recognized as a signal peptide and that positioning it at the N terminus interferes with PEXEL processing (Fig. 4C). Indeed, while the mNG signal within parasites expressing the WT reporter localized to the digestive vacuole (typical for proteins secreted to the PV or exported into the host cell, which are partially endocytosed along with host cell cytosol), the mNG signal within parasites expressing the truncated reporter was also perinuclear and showed a significant increase in co-localization with ER tracker, suggesting that the repositioned signal sequence did not support efficient ER exit (Fig. 4B and D). Taken together, this indicates that the typical recessed position of the signal sequence in exported PEXEL proteins is important for PEXEL processing but is fully exchangeable with a classical signal peptide configuration at the extreme N terminus for efficient PEXEL processing, suggesting that different modes of ER entry can support PEXEL maturation.

## DISCUSSION

The discovery of the PEXEL motif revolutionized the identification of host-targeted effector proteins in *Plasmodium* due to its high predictive power for export (8, 9, 20, 27–29). Consequently, PEXEL processing is generally thought to be restricted to exported proteins. In this study, we reveal PEXEL processing also occurs in the non-exported PV and rhoptry proteins UIS2, RON11, and PMIX. While deviations at the strongly constrained P3, P1, and P2′ positions have been shown to support processing and export in certain contexts (29), the flexibility of residues occupying these positions is limited (14, 18, 20–22, 25). In contrast, a wide range of charge-neutral residues can occupy P2 and P1′. While successful PEXEL cleavage generally leads to protein export, the P1′ residue, which becomes the N-terminal most position in the mature N terminus, can interfere with export after processing (19, 21, 28). Remarkably, we found that the inability of the mature N termini of UIS2 and RON11 to mediate export is attributable to this single amino acid (aspartic acid or phenylalanine) at position P1′ in the PEXEL of these proteins. Notably, the UIS2 PEXEL motif differs between *P. berghei* and *P. falciparum* (RVLQE vs RILDE), indicating that glutamic acid at P1′ likely also inhibits export. Collectively, this suggests that PEXEL P1′ residues with charged or certain bulky side chains (phenylalanine but not tyrosine) are competent for processing but not export. Consistent with this, a recent report indicates lysine is also not tolerated for export at P1′ (42). For PV resident proteins that do not function in the host cell, these P1′ residues would provide a control mechanism to allow for proteolytic maturation by PMV without risking unproductive export, presumably preventing recognition by the PTEX unfoldase HSP101 (43).

While most PEXEL proteins are synthesized after merozoite invasion and trafficked directly to the PV for export, some are packaged into dense granules (RESA and LSA3) during merozoite formation and secreted from this organelle into the forming PV for export immediately upon invasion (20, 44, 45). A surprising finding of our study is the presence of cleavable PEXELs in the rhoptry proteins RON11 and PMIX, indicating that PEXEL processing is a more general maturation event than previously appreciated. While the P1′ of RON11 prevents export, similar to UIS2, PMIX encodes a serine at position P1′, which is found in the PEXEL of many exported proteins (8, 9, 27) and processing of the PMIX PEXEL did result in reporter export. Exported proteins follow the default secretory pathway to the PV, while proteins that traffic to other destinations contain targeting information that overrides default PV secretion (46, 47). This suggests that the dominant rhoptry trafficking signal in PMIX prevents it from reaching the PV and being exported. While unfolding and PVM translocation of exported proteins occurs in the vacuole, it should be noted that HSP101 has recently been proposed to recognize cargo upstream in the ER (22). However, PMIX undergoes an additional autocatalytic maturation step (p73 > p55) that removes a further ~145 residues after the mature PEXEL (predicted cleavage site NT↓SQ where S194 becomes the N-terminal most residue in p55) (37, 38, 48), eliminating the mature PEXEL N terminus that might otherwise drive the export of the protease. As no N-terminal tagging has been applied to PMIX, it is not clear whether this cleaved fragment is ultimately exported beyond the parasite. Notably, while the RON11 PEXEL is widely conserved in orthologs from other *Plasmodium* spp., the PMIX PEXEL is not (Fig. S4). The functional significance of PEXEL processing in PfPMIX and closely related *Laverania* species is unclear, but lack of PEXEL conservation implies that initial cleavage by PMV is not generally important for activating PMIX to enable its ultimate autocatalytic maturation.

Entry into the secretory pathway via a signal peptide or transmembrane domain is mediated by the SEC61 complex which, in *Plasmodium*, appears to form distinct associations with either signal peptidase or PMV (10). While exported PEXEL proteins often contain a recessed signal sequence thought to act as a membrane anchor that is removed by PMV, UIS2 and RON11 encode a classical signal peptide, similar to many members of the STEVOR and RIFIN families, suggesting that multiple ER entry points are tolerated en route to PMV. If that were the case, then repositioning the recessed signal sequence domain of exported proteins in a conformation that mimics a classical signal peptide should still route these proteins to PMV through the signal peptidase-SEC61 complex. While a portion of the truncated reporter was still exported, this configuration resulted in increased ER retention with a major reduction in PEXEL processing, indicating that the repositioned signal sequence mediates insertion into the ER membrane but is not efficiently recognized and cleaved by either signal peptidase or PMV. Notably, several PEXEL mutants that block PMV processing accumulate as the unprocessed, full-length form of the protein, further suggesting that the recessed signal sequence is not efficiently recognized as a signal peptide (10, 12–14, 18, 20, 29). As the EMP3∆2–11 construct preserves the spacing between the signal sequence and PEXEL that is important for cleavage by PMV (14), inefficient processing might alternatively indicate altered topology where the truncated protein is partially inserted into the ER membrane as a type III transmembrane protein, placing the C-terminus in the cytoplasm where it is inaccessible to PMV (49). Curiously, P3 R > A mutations that blocked PEXEL processing in RON11 and UIS2 accumulated as two distinct species, suggesting that the signal peptide in these proteins is not efficiently recognized by signal peptidase and may act as a stable signal anchor similar to PEXEL proteins encoding recessed signal sequences. If this is the case, then PEXEL-containing proteins may also enter the ER through the PMV-SEC61 complex regardless of signal sequence positioning.

The discovery of PEXEL processing in non-exported proteins may indicate that PMV was historically a more general secretory maturase for which many additional substrates have evolved through an increase in the number of exported proteins that contain a PEXEL, particularly in expanded families such as RIFINs and PHISTs in *P. falciparum* (27). As the parasite became heavily dependent on protein export, the PMV-SEC61 complex may have been adapted to alleviate the burden on the canonical SEC61 complex. Of note, a similar *Toxoplasma* export element (TEXEL, RRLxx) is cleaved by the PMV ortholog ASP5. Although TEXEL processing is also important for vacuolar export of *Toxoplasma* effector proteins (50–52), many ASP5 substrates are PV-resident proteins that are not translocated into the host cell (53) and processing has been shown to be dispensable for localization and function of some non-exported proteins (54). In apparent contrast to *Plasmodium*, TEXEL processing is not restricted to the N terminus, and some proteins are processed at multiple TEXELs, such as the UIS2 ortholog GRA44 (54–56). C-terminally located PEXEL sequences can also be found in *Plasmodium* proteins but are not known to be processed. For instance, ClpB1, a nuclear-encoded HSP100 that is targeted to the apicoplast, contains an identical PEXEL to PfUIS2 (RILDE) located at positions 1,044–1,048 of 1,070; however, this does not appear to be appreciably processed as C-terminal epitope tags are retained on the full length protein, even when it is artificially retrieved to the ER (57).

Curiously, ASP5 is located in the Golgi (58), suggesting that cargo recognition of exported ASP5 substrates functions differently in *Toxoplasma gondii*. Indeed, *T. gondii* possess an analogous but mechanistically different vacuolar export pathway that depends upon the MYR complex thought to form a distinct translocon (56, 59, 60). That PEXEL processing occurs coincidentally with ER entry in *Plasmodium* suggests that the mature, export-mediating N terminus may be recognized early in the secretory pathway, presumably by HSP101, although it is unclear why this should occur upstream of the site of membrane translocation by PTEX (22). Future studies that determine why certain mature PEXEL N termini enable selection for PVM translocation will be important to resolve the connection between these key events at opposite ends of the parasite secretory pathway.

## MATERIALS AND METHODS

### Parasite culture

*P. falciparum* NF54^attB-DiCre^ (57) and derivatives were cultured in RPMI 1640 medium supplemented with 27 mM sodium bicarbonate, 11 mM glucose, 0.37 mM hypoxanthine, 10 µg/mL gentamicin, and 0.5% Albumax I (Gibco). Parasites were maintained in deidentified, Institutional Review Board-exempt RBCs obtained from the American National Red Cross.

### Plasmids and genetic modification of *P. falciparum*

All cloning was carried out with NEBuilder HIFI DNA assembly (NEB). Primer sequences are given in Table S1. For Cpf1-mediated editing of the UIS2 locus, the pAIO-LbCpf1 plasmid (35) was modified by inserting a HindIII site adjacent to the AflII site in the pre-gRNA cassette using primer P1, resulting in the plasmid JRB489, which enables double digestion to reduce parent vector background during cloning. To target the 3′ end of *uis2*, a Cpf1 gRNA target was chosen just downstream of the stop codon (TATTACCTTGATATTCTATTAAGG), and the gRNA seed sequence was synthesized in primer P2 and inserted at HindIII/AflII in JRB489, resulting in plasmid JRB484. To generate UIS2-mNG parasites, a 5′ homology flank (up to but not including the stop codon) and 3′ homology flank (beginning immediately downstream of the Cpf1 gRNA PAM) were amplified from genomic DNA using P3/4 and P5/6, respectively, assembled in a second PCR reaction using P4/5 and inserted between XhoI/AvrII in JRB508 (57), resulting in plasmid JRB509. This plasmid was linearized with AflII and co-transfected with JRB484 into NF54^attB-DiCre^. Selection was applied with 5 nM WR99120 24 h post-transfection, and clonal lines were isolated by limiting dilution after parasites returned from the selection.

To generate the UIS2_1-70_-mNG fusion cassette, the 5′ cds of *uis2* encoding the first 70 amino acids was amplified from gDNA using P7/8 and inserted at AvrII in JRB416 (57) removing the adjacent loxP site and inserting an NheI site between the *uis2* and mNG sequences, resulting in plasmid JRB510. An R43A PEXEL mutation in UIS2_1-70_-mNG was generated in this plasmid using a QuikChange Lightning Multi Site Directed Mutagenesis kit (Agilent) and the primer P9, resulting in plasmid JRB525. A D46A PEXEL mutation in UIS2_1-70_-mNG was generated using primers P7/31 and P32/P8 to amplify two PCR products that were then stitched together in a second PCR reaction using primers P7/8 resulting in plasmid MAF63. To generate the UIS2-EMP3 chimeric constructs, primer pairs were used to amplify the leader sequence of UIS2 including the endogenous PEXEL (P7/P10), an R43A PEXEL mutation (P7/P11), a D46A PEXEL mutation (P7/33), and replacing the UIS2 PEXEL with the EMP3 PEXEL (P7/P12), while appending codons 65–74 of EMP3. A second PCR reaction was performed using this initial PCR amplicon as a template with primers P7/13 to append codons 75–82 of EMP3, which was subsequently inserted into JRB510 between AvrII/NheI generating plasmids MAF45, MAF52, MAF61, and MAF53, respectively. A similar approach was used to generate UIS2-A12 chimeras where primer pairs were used to amplify the leader sequence of UIS2 including the endogenous PEXEL (P7/P14), R43A PEXEL mutation (P7/P15), and an R46A PEXEL mutation (P7/P16) while appending 12 alanine codons after the PEXEL. These were inserted into JRB510 between AvrII/NheI generating plasmids MAF48, MAF59, and MAF57, respectively. These plasmids were co-transfected with pINT (61) into NF54^attB-DiCre^ parasites and selection was applied with 2.5 µg/mL blasticidin-S 48 h post-transfection.

To generate the EMP3-mNG fusion construct, primers P17, P18, P19, and P13 were used to synthetically generate codons 1–82 of EMP3. Using 10 µM of each primer, an initial PCR reaction was performed using the following parameters: 94°C for 5 min (94°C for 2 min, 60°C for 2 min, and 68°C for 3 min) × nine rounds, 68°C for 5 min, 4°C ∞. One microliter of this initial reaction was then used in a subsequent, standard PCR reaction using primers P17/P13, which was then inserted into JRB510 between AvrII/NheI generating plasmid MAF51. This same construct was regenerated to remove a residual loxP site found after the HA tag by digesting with AvrII/AflII. Primers P17/20 were then used to re-amplify the EMP3-mNG fusion, which was then inserted between AvrII/AflII generating plasmid MAF64. To generate EMP3-mNG without the recessed signal peptide, a similar approach was used except using primers P21, P18, P19, and P13 in an initial PCR reaction and then using primers P21/P13 to generate the final product inserted into JRB510 between AvrII/NheI generating plasmid MAF54. These plasmids were co-transfected with pINT into NF54^attB-DiCre^ parasites, and selection was applied with 2.5 µg/mL blasticidin-S 48 h post-transfection.

To generate the RON11-mNG fusion cassette, primers P22/P23 were used to amplify codons 1–70 of RON11, while removing the N-terminal intron, and inserted into JRB510 between AvrII/NheI generating plasmid MAF47. To generate the R51A or F54A mutation of the RON11 PEXEL, plasmid MAF47 was linearized with the site BstBI that resides within the PEXEL, and primer P24 or P25, containing the PEXEL mutation, was used to seal the vector using NEBuilder HIFI DNA assembly, resulting in plasmids MAF50 and MAF60. To generate the PMIX-mNG fusion cassette, primers P26/P27 were used to amplify codons 1–71 of PMIX and inserted into JRB510 between AvrII/NheI generating plasmid MAF46. To generate the R46A PEXEL mutation, primers P26, P28, P29, and P30 were used to synthetically generate codons 1–71 of PMIX with the PEXEL mutation in an initial PCR reaction, as described above, followed by a second PCR reaction using primers P26/P27, which was then inserted into JRB510 between AvrII/NheI generating plasmid MAF49.

### Microscopy and analysis

For live microscopy, Hoechst (33342, Invitrogen; 1:10,000) was used to visualize the nucleus by incubating with parasites 2–5 min prior to visualization. ER-Tracker Red (Invitrogen; 1:1000) was used to visualize the ER by incubating with parasites for 30 min at 37°C prior to visualization. Parasites were imaged on an Axio Observer 7 equipped with an Axiocam 702 mono camera and Zen 2.6 Pro software (Zeiss) using the same exposure times for all images across sample groups and experimental replicates. Image processing, analysis, and display were performed using Zeiss Blue software and Adobe Photoshop. Equivalent adjustments to brightness and contrast were made within sample groups for display purposes. Quantification of export for reporters presented in Fig. 1 to 3; Fig. S2B through F was calculated by categorizing mNG localization on a per cell basis as strictly in the PV/parasite vs exported into the RBC. For the quantification of export for the reporters presented in Fig. 4, mNG-integrated intensity in the RBC cytosol was calculated by creating an ROI corresponding to the PV/parasite or the entire RBC using the DIC channel as a guide, collecting the integrated density within these regions and then subtracting the integrated density within the PV/parasite from the integrated density within the entire RBC; integrated density measurements were acquired using Fiji (62). Pearson’s correlation was calculated using Cell Profiler (version 4.2.6) (41).

### Protease protection assay

Protease protection assays were adapted from reference (57). Briefly, late-stage parasites were magnet purified and washed with 1× phosphate-buffered saline (PBS). Then, 5 hemolytic units (HUs) of Streptolysin-O (SLO, Millipore-Sigma; SAE0089) [for activation, storage, and determining a HU, see reference (63)] was used to permeabilize the RBC membrane for 6 min at room temperature. SLO was removed by washing once with 1 mL of 1× PBS and once with 1 mL of Digestion Buffer (50 mM Tris pH 7.5, 150 mM NaCl, and 1 mM CaCl_2_). Samples were then split into SLO-only treated samples that were subsequently treated with or without Proteinase K (Qiagen, 19131) for 15 min at 37°C or further treated with ice-cold saponin for 10 min before subsequent treatment with Proteinase K. After treatment, parasites were stained with Hoechst 34580 (Invitrogen, H21486), and the mean mNeonGreen fluorescence was collected on an Attune NxT flow cytometer (ThermoFisher) by gating on parasites using Hoechst fluorescence and then collecting the mNG MFI from this population.

### Western blotting

Western blotting for *Plasmodium* parasites was performed as described previously (35, 64). Briefly, parasites were selectively permeabilized by treatment with ice-cold 0.03% saponin in PBS for 15 min followed by lysis with RIPA to remove the hemozoin. The antibodies used in this study were mouse anti-HA antibody (clone 16B12, BioLegend; 1:500), rabbit polyclonal anti-HA SG77 (ThermoFisher; 1:500), mouse monoclonal anti-FLAG clone M2 (Sigma-Aldrich; 1:200), and mouse monoclonal anti-EXP2 clone 7.7 (1:500). The secondary antibodies used are IRDye 680CW goat anti-mouse IgG (LICOR Biosciences) (1:20,000). Western blot images were processed using the Odyssey Clx LICOR infrared imaging system software (LICOR Biosciences). Full-length blots are presented in Fig. S5.

### LC-MS acquisition and analysis

For sample preparation of parasite lysates, see reference (35). Peptide samples were separated on a 75 µM ID × 25 cm C18 column packed with 1.9 µm C18 particles (Dr. Maisch GmbH) using a 140-min gradient of increasing acetonitrile and eluted directly into a Thermo Orbitrap Fusion Lumos mass spectrometer, where MS spectra were acquired by data-dependent acquisition. Data were searched using MaxQuant and a customized *Plasmodium falciparum 3D7* database containing the predicted N-terminally processed version of PF3D7_1464600 with carbamidomethylation set as a fixed modification and methionine oxidation and N-terminal acetylation set as variable modifications. The digestion mode was set to trypsin with a maximum of two missed cleavages. Precursor mass tolerances of 20 and 4.5 ppm were applied to the first and second searches, respectively, while a 20 ppm mass tolerance was used for fragment ions. The data sets were filtered at a 1% false discovery rate at both the peptide-spectrum match and protein levels.

## SUPPLEMENTAL MATERIAL

Supplemental figures and tables msphere.00393-23-s0001.pdf Figures S1-S5 and Table S1.

ASM does not own the copyrights to Supplemental Material that may be linked to, or accessed through, an article. The authors have granted ASM a non-exclusive, world-wide license to publish the Supplemental Material files. Please contact the corresponding author directly for reuse.

## References

[1] Jonsdottir TK, Gabriela M, Crabb BS, F de Koning-Ward T, Gilson PR. 2021. Defining the essential exportome of the malaria parasite. Trends Parasitol 37:664–675. doi:10.1016/j.pt.2021.04.009 33985912

[2] Matz JM, Beck JR, Blackman MJ. 2020. The parasitophorous vacuole of the blood-stage malaria parasite. Nat Rev Microbiol 18:379–391. doi:10.1038/s41579-019-0321-3 31980807

[3] Gehde N, Hinrichs C, Montilla I, Charpian S, Lingelbach K, Przyborski JM. 2009. Protein unfolding is an essential requirement for transport across the parasitophorous vacuolar membrane of Plasmodium falciparum. Mol Microbiol 71:613–628. doi:10.1111/j.1365-2958.2008.06552.x 19040635

[4] de Koning-Ward TF, Gilson PR, Boddey JA, Rug M, Smith BJ, Papenfuss AT, Sanders PR, Lundie RJ, Maier AG, Cowman AF, Crabb BS. 2009. A newly discovered protein export machine in malaria parasites. Nature 459:945–949. doi:10.1038/nature08104 19536257 PMC2725363

[5] Beck JR, Muralidharan V, Oksman A, Goldberg DE. 2014. PTEX component HSP101 mediates export of diverse malaria effectors into host erythrocytes. Nature 511:592–595. doi:10.1038/nature13574 25043010 PMC4130291

[6] Elsworth B, Matthews K, Nie CQ, Kalanon M, Charnaud SC, Sanders PR, Chisholm SA, Counihan NA, Shaw PJ, Pino P, Chan J-A, Azevedo MF, Rogerson SJ, Beeson JG, Crabb BS, Gilson PR, de Koning-Ward TF. 2014. PTEX is an essential nexus for protein export in malaria parasites. Nature 511:587–591. doi:10.1038/nature13555 25043043

[7] Mesén-Ramírez P, Reinsch F, Blancke Soares A, Bergmann B, Ullrich A-K, Tenzer S, Spielmann T. 2016. Stable translocation intermediates jam global protein export in Plasmodium falciparum parasites and link the PTEX component EXP2 with translocation activity. PLoS Pathog 12:e1005618. doi:10.1371/journal.ppat.1005618 27168322 PMC4864081

[8] Marti M, Good RT, Rug M, Knuepfer E, Cowman AF. 2004. Targeting malaria virulence and remodeling proteins to the host erythrocyte. Science 306:1930–1933. doi:10.1126/science.1102452 15591202

[9] Hiller NL, Bhattacharjee S, van Ooij C, Liolios K, Harrison T, Lopez-Estraño C, Haldar K. 2004. A host-targeting signal in virulence proteins reveals a secretome in malarial infection. Science 306:1934–1937. doi:10.1126/science.1102737 15591203

[10] Marapana DS, Dagley LF, Sandow JJ, Nebl T, Triglia T, Pasternak M, Dickerman BK, Crabb BS, Gilson PR, Webb AI, Boddey JA, Cowman AF. 2018. Plasmepsin V cleaves malaria effector proteins in a distinct endoplasmic reticulum translocation interactome for export to the erythrocyte. Nat Microbiol 3:1010–1022. doi:10.1038/s41564-018-0219-2 30127496

[11] Russo I, Babbitt S, Muralidharan V, Butler T, Oksman A, Goldberg DE. 2010. Plasmepsin V licenses Plasmodium proteins for export into the host erythrocyte. Nature 463:632–636. doi:10.1038/nature08726 20130644 PMC2826791

[12] Boddey JA, Hodder AN, Günther S, Gilson PR, Patsiouras H, Kapp EA, Pearce JA, de Koning-Ward TF, Simpson RJ, Crabb BS, Cowman AF. 2010. An aspartyl protease directs malaria effector proteins to the host cell. Nature 463:627–631. doi:10.1038/nature08728 20130643 PMC2818761

[13] Sleebs BE, Lopaticki S, Marapana DS, O’Neill MT, Rajasekaran P, Gazdik M, Günther S, Whitehead LW, Lowes KN, Barfod L, Hviid L, Shaw PJ, Hodder AN, Smith BJ, Cowman AF, Boddey JA. 2014. Inhibition of plasmepsin V activity demonstrates its essential role in protein export, PfEMP1 display, and survival of malaria parasites. PLoS Biol 12:e1001897. doi:10.1371/journal.pbio.1001897 24983235 PMC4077696

[14] Boddey JA, O’Neill MT, Lopaticki S, Carvalho TG, Hodder AN, Nebl T, Wawra S, van West P, Ebrahimzadeh Z, Richard D, Flemming S, Spielmann T, Przyborski J, Babon JJ, Cowman AF. 2016. Export of malaria proteins requires co-translational processing of the PEXEL motif independent of phosphatidylinositol-3-phosphate binding. Nat Commun 7:10470. doi:10.1038/ncomms10470 26832821 PMC4740378

[15] Chang HH, Falick AM, Carlton PM, Sedat JW, DeRisi JL, Marletta MA. 2008. N-terminal processing of proteins exported by malaria parasites. Mol Biochem Parasitol 160:107–115. doi:10.1016/j.molbiopara.2008.04.011 18534695 PMC2922945

[16] Nyonda MA, Boyer J-B, Belmudes L, Krishnan A, Pino P, Couté Y, Brochet M, Meinnel T, Soldati-Favre D, Giglione C. 2022. N-acetylation of secreted proteins in apicomplexa is widespread and is independent of the ER acetyl-CoA transporter AT1. J Cell Sci 135:jcs259811. doi:10.1242/jcs.259811 35621049

[17] Polino AJ, Hasan MM, Floyd K, Avila-Cruz Y, Yang Y, Goldberg DE. 2023. An essential endoplasmic reticulum-resident N-acetyltransferase ortholog in Plasmodium falciparum. J Cell Sci 136:jcs260551. doi:10.1242/jcs.260551 36744402 PMC10038149

[18] Boddey J. A., Moritz RL, Simpson RJ, Cowman AF. 2009. Role of the Plasmodium export element in trafficking parasite proteins to the infected erythrocyte. Traffic 10:285–299. doi:10.1111/j.1600-0854.2008.00864.x 19055692 PMC2682620

[19] Grüring C, Heiber A, Kruse F, Flemming S, Franci G, Colombo SF, Fasana E, Schoeler H, Borgese N, Stunnenberg HG, Przyborski JM, Gilberger T-W, Spielmann T. 2012. Uncovering common principles in protein export of malaria parasites. Cell Host Microbe 12:717–729. doi:10.1016/j.chom.2012.09.010 23159060

[20] Boddey JA, Carvalho TG, Hodder AN, Sargeant TJ, Sleebs BE, Marapana D, Lopaticki S, Nebl T, Cowman AF. 2013. Role of plasmepsin V in export of diverse protein families from the Plasmodium falciparum exportome. Traffic 14:532–550. doi:10.1111/tra.12053 23387285

[21] Tarr SJ, Cryar A, Thalassinos K, Haldar K, Osborne AR. 2013. The C-terminal portion of the cleaved HT motif is necessary and sufficient to mediate export of proteins from the malaria parasite into its host cell. Mol Microbiol 87:835–850. doi:10.1111/mmi.12133 23279267 PMC3567231

[22] Gabriela M, Matthews KM, Boshoven C, Kouskousis B, Jonsdottir TK, Bullen HE, Modak J, Steer DL, Sleebs BE, Crabb BS, de Koning-Ward TF, Gilson PR. 2022. A revised mechanism for how Plasmodium falciparum recruits and exports proteins into its erythrocytic host cell. PLoS Pathog 18:e1009977. doi:10.1371/journal.ppat.1009977 35192672 PMC8896661

[23] Hasan MM, Polino AJ, Mukherjee S, Vaupel B, Goldberg DE, Miller LH. 2023. The mature N-termini of Plasmodium effector proteins confer specificity of export . mBio 14. doi:10.1128/mbio.01215-23 PMC10653839 37646514

[24] Haase S, Herrmann S, Grüring C, Heiber A, Jansen PW, Langer C, Treeck M, Cabrera A, Bruns C, Struck NS, Kono M, Engelberg K, Ruch U, Stunnenberg HG, Gilberger T-W, Spielmann T. 2009. Sequence requirements for the export of the Plasmodium falciparum maurer’s clefts protein REX2. Mol Microbiol 71:1003–1017. doi:10.1111/j.1365-2958.2008.06582.x 19170882

[25] Gabriela M, Barnes CBG, Leong D, Sleebs BE, Schneider MP, Littler DR, Crabb BS, de Koning-Ward TF, Gilson PR. 2023. Sequence elements within the PEXEL motif and its downstream region modulate PTEX-dependent protein export in Plasmodium falciparum. Traffic 10. doi:10.1111/tra.12922 PMC10952997 37926971

[26] Heiber A, Kruse F, Pick C, Grüring C, Flemming S, Oberli A, Schoeler H, Retzlaff S, Mesén-Ramírez P, Hiss JA, Kadekoppala M, Hecht L, Holder AA, Gilberger T-W, Spielmann T. 2013. Identification of new PNEPs indicates a substantial non-PEXEL exportome and underpins common features in Plasmodium falciparum protein export. PLoS Pathog 9:e1003546. doi:10.1371/journal.ppat.1003546 23950716 PMC3738491

[27] Sargeant TJ, Marti M, Caler E, Carlton JM, Simpson K, Speed TP, Cowman AF. 2006. Lineage-specific expansion of proteins exported to erythrocytes in malaria parasites. Genome Biol 7:R12. doi:10.1186/gb-2006-7-2-r12 16507167 PMC1431722

[28] van Ooij C, Tamez P, Bhattacharjee S, Hiller NL, Harrison T, Liolios K, Kooij T, Ramesar J, Balu B, Adams J, Waters A, Janse C, Haldar K, Kazura J. 2008. The malaria secretome: from algorithms to essential function in blood stage infection. PLoS Pathog 4:e1000084. doi:10.1371/journal.ppat.1000084 18551176 PMC2408878

[29] Schulze J, Kwiatkowski M, Borner J, Schlüter H, Bruchhaus I, Burmester T, Spielmann T, Pick C. 2015. The Plasmodium falciparum exportome contains non-canonical PEXEL/HT proteins. Mol Microbiol 97:301–314. doi:10.1111/mmi.13024 25850860

[30] Shears MJ, Sekhar Nirujogi R, Swearingen KE, Renuse S, Mishra S, Jaipal Reddy P, Moritz RL, Pandey A, Sinnis P. 2019. Proteomic analysis of Plasmodium merosomes: the link between liver and blood stages in malaria. J Proteome Res 18:3404–3418. doi:10.1021/acs.jproteome.9b00324 31335145 PMC7105274

[31] McConville R, Krol J, Steel R, O’Neill M, Davey B, Hodder A, Nebl T, Cowman A, Kneteman NM, Boddey J. 2023. *Plasmodium falciparum* exoerythrocytic forms require the PTEX translocon for development in human hepatocytes. In review. Research Square Platform LLC. doi:10.21203/rs.3.rs-2632356/v1

[32] Khosh-Naucke M, Becker J, Mesén-Ramírez P, Kiani P, Birnbaum J, Fröhlke U, Jonscher E, Schlüter H, Spielmann T. 2018. Identification of novel parasitophorous vacuole proteins in P. falciparum parasites using Bioid. Int J Med Microbiol 308:13–24. doi:10.1016/j.ijmm.2017.07.007 28784333

[33] Schnider CB, Bausch-Fluck D, Brühlmann F, Heussler VT, Burda P-C. 2018. Bioid reveals novel proteins of the Plasmodium parasitophorous vacuole membrane. mSphere 3:e00522-17. doi:10.1128/mSphere.00522-17 PMC5784244 29404413

[34] Zhang M, Mishra S, Sakthivel R, Fontoura BMA, Nussenzweig V. 2016. UIS2: a unique phosphatase required for the development of Plasmodium liver stages. PLoS Pathog 12:e1005370. doi:10.1371/journal.ppat.1005370 26735921 PMC4712141

[35] Nessel T, Beck JM, Rayatpisheh S, Jami-Alahmadi Y, Wohlschlegel JA, Goldberg DE, Beck JR. 2020. EXP1 is required for organisation of EXP2 in the intraerythrocytic malaria parasite vacuole. Cell Microbiol 22:e13168. doi:10.1111/cmi.13168 31990132 PMC7138706

[36] Przyborski JM, Miller SK, Pfahler JM, Henrich PP, Rohrbach P, Crabb BS, Lanzer M. 2005. Trafficking of STEVOR to the Maurer’s clefts in Plasmodium falciparum-infected erythrocytes. EMBO J 24:2306–2317. doi:10.1038/sj.emboj.7600720 15961998 PMC1173160

[37] Nasamu AS, Glushakova S, Russo I, Vaupel B, Oksman A, Kim AS, Fremont DH, Tolia N, Beck JR, Meyers MJ, Niles JC, Zimmerberg J, Goldberg DE. 2017. Plasmepsins IX and X are essential and druggable mediators of malaria parasite egress and invasion. Science 358:518–522. doi:10.1126/science.aan1478 29074774 PMC5928414

[38] Pino P, Caldelari R, Mukherjee B, Vahokoski J, Klages N, Maco B, Collins CR, Blackman MJ, Kursula I, Heussler V, Brochet M, Soldati-Favre D. 2017. A multistage antimalarial targets the plasmepsins IX and X essential for invasion and egress. Science 358:522–528. doi:10.1126/science.aaf8675 29074775 PMC5730047

[39] Bantuchai S, Nozaki M, Thongkukiatkul A, Lorsuwannarat N, Tachibana M, Baba M, Matsuoka K, Tsuboi T, Torii M, Ishino T. 2019. Rhoptry neck protein 11 has crucial roles during malaria parasite sporozoite invasion of salivary glands and hepatocytes. Int J Parasitol 49:725–735. doi:10.1016/j.ijpara.2019.05.001 31247198

[40] Bendtsen JD, Nielsen H, von Heijne G, Brunak S. 2004. Improved prediction of signal peptides: signalP 3.0. J Mol Biol 340:783–795. doi:10.1016/j.jmb.2004.05.028 15223320

[41] Stirling DR, Swain-Bowden MJ, Lucas AM, Carpenter AE, Cimini BA, Goodman A. 2021. CellProfiler 4: improvements in speed, utility and usability. BMC Bioinformatics 22:433. doi:10.1186/s12859-021-04344-9 34507520 PMC8431850

[42] Fréville A, Ressurreição M, van Ooij C. 2024. Identification of a non-exported plasmepsin V substrate that functions in the parasitophorous vacuole of malaria parasites. mBio 15:e0122323. doi:10.1128/mbio.01223-23 38078758 PMC10790765

[43] Ho C-M, Beck JR, Lai M, Cui Y, Goldberg DE, Egea PF, Zhou ZH. 2018. Malaria parasite translocon structure and mechanism of effector export. Nature 561:70–75. doi:10.1038/s41586-018-0469-4 30150771 PMC6555636

[44] Aikawa M, Torii M, Sjölander A, Berzins K, Perlmann P, Miller LH. 1990. Pf155/RESA antigen is localized in dense granules of Plasmodium-falciparum merozoites. Exp Parasitol 71:326–329. doi:10.1016/0014-4894(90)90037-d 2209790

[45] Morita M, Takashima E, Ito D, Miura K, Thongkukiatkul A, Diouf A, Fairhurst RM, Diakite M, Long CA, Torii M, Tsuboi T. 2017. Immunoscreening of Plasmodium falciparum proteins expressed in a wheat germ cell-free system reveals a novel malaria vaccine candidate. Sci Rep 7:46086. doi:10.1038/srep46086 28378857 PMC5380959

[46] Waller RF, Reed MB, Cowman AF, McFadden GI. 2000. Protein trafficking to the plastid of Plasmodium falciparum is via the secretory pathway. EMBO J 19:1794–1802. doi:10.1093/emboj/19.8.1794 10775264 PMC302007

[47] Adisa A, Rug M, Klonis N, Foley M, Cowman AF, Tilley L. 2003. The signal sequence of exported protein-1 directs the green fluorescent protein to the parasitophorous vacuole of transfected malaria parasites. J Biol Chem 278:6532–6542. doi:10.1074/jbc.M207039200 12456681

[48] Favuzza P, de Lera Ruiz M, Thompson JK, Triglia T, Ngo A, Steel RWJ, Vavrek M, Christensen J, Healer J, Boyce C, et al.. 2020. Dual plasmepsin-targeting antimalarial agents disrupt multiple stages of the malaria parasite life cycle. Cell Host Microbe 27:642–658. doi:10.1016/j.chom.2020.02.005 32109369 PMC7146544

[49] Tarr SJ, Osborne AR. 2015. Experimental determination of the membrane topology of the Plasmodium protease plasmepsin V. PLoS One 10:e0121786. doi:10.1371/journal.pone.0121786 25849462 PMC4388684

[50] Coffey MJ, Sleebs BE, Uboldi AD, Garnham A, Franco M, Marino ND, Panas MW, Ferguson DJ, Enciso M, O’Neill MT, Lopaticki S, Stewart RJ, Dewson G, Smyth GK, Smith BJ, Masters SL, Boothroyd JC, Boddey JA, Tonkin CJ. 2015. An aspartyl protease defines a novel pathway for export of Toxoplasma proteins into the host cell. Elife 4:e10809. doi:10.7554/eLife.10809 26576949 PMC4764566

[51] Curt-Varesano A, Braun L, Ranquet C, Hakimi MA, Bougdour A. 2016. The aspartyl protease TgASP5 mediates the export of the Toxoplasma GRA16 and GRA24 effectors into host cells. Cell Microbiol 18:151–167. doi:10.1111/cmi.12498 26270241

[52] Hammoudi P-M, Jacot D, Mueller C, Di Cristina M, Dogga SK, Marq J-B, Romano J, Tosetti N, Dubrot J, Emre Y, Lunghi M, Coppens I, Yamamoto M, Sojka D, Pino P, Soldati-Favre D. 2015. Fundamental roles of the Golgi-associated Toxoplasma aspartyl protease, ASP5, at the host-parasite interface. PLoS Pathog 11:e1005211. doi:10.1371/journal.ppat.1005211 26473595 PMC4608785

[53] Hsiao C-HC, Luisa Hiller N, Haldar K, Knoll LJ. 2013. A HT/PEXEL motif in Toxoplasma dense granule proteins is a signal for protein cleavage but not export into the host cell. Traffic 14:519–531. doi:10.1111/tra.12049 23356236 PMC3622808

[54] Coffey MJ, Dagley LF, Seizova S, Kapp EA, Infusini G, Roos DS, Boddey JA, Webb AI, Tonkin CJ. 2018. Aspartyl protease 5 matures dense granule proteins that reside at the host-parasite interface in Toxoplasma gondii. mBio 9:e01796-18. doi:10.1128/mBio.01796-18 30377279 PMC6212819

[55] Blakely WJ, Holmes MJ, Arrizabalaga G. 2020. The secreted acid phosphatase domain-containing GRA44 from Toxoplasma gondii is required for c-Myc induction in infected cells. mSphere 5:e00877-19. doi:10.1128/mSphere.00877-19 32075881 PMC7031617

[56] Cygan AM, Theisen TC, Mendoza AG, Marino ND, Panas MW, Boothroyd JC. 2020. Coimmunoprecipitation with Myr1 identifies three additional proteins within the Toxoplasma gondii parasitophorous vacuole required for translocation of dense granule effectors into host cells. mSphere 5:e00858-19. doi:10.1128/mSphere.00858-19 PMC7031616 32075880

[57] Fierro MA, Hussain T, Campin LJ, Beck JR. 2023. Knock-sideways by inducible ER retrieval enables a unique approach for studying Plasmodium-secreted proteins. Proc Natl Acad Sci U S A 120:e2308676120. doi:10.1073/pnas.2308676120 37552754 PMC10433460

[58] Shea M, Jäkle U, Liu Q, Berry C, Joiner KA, Soldati-Favre D. 2007. A family of aspartic proteases and a novel, dynamic and cell-cycle-dependent protease localization in the secretory pathway of Toxoplasma gondii. Traffic 8:1018–1034. doi:10.1111/j.1600-0854.2007.00589.x 17547703

[59] Franco M, Panas MW, Marino ND, Lee M-CW, Buchholz KR, Kelly FD, Bednarski JJ, Sleckman BP, Pourmand N, Boothroyd JC. 2016. A novel secreted protein, MYR1, is central to Toxoplasma’s manipulation of host cells. mBio 7:e02231–15. doi:10.1128/mBio.02231-15 26838724 PMC4742717

[60] Marino ND, Panas MW, Franco M, Theisen TC, Naor A, Rastogi S, Buchholz KR, Lorenzi HA, Boothroyd JC. 2018. Identification of a novel protein complex essential for effector translocation across the parasitophorous vacuole membrane of Toxoplasma gondii. PLoS Pathog 14:e1006828. doi:10.1371/journal.ppat.1006828 29357375 PMC5794187

[61] Braman J. 2010. *In vitro* mutagenesis protocols. In A method for rapid genetic integration into Plasmodium falciparum utilizing mycobacteriophage BXB1 Integrase. Humana Press, Totowa, NJ. 10.1007/978-1-60761-652-8_6 PMC3119856 20676977

[62] Schindelin J, Arganda-Carreras I, Frise E, Kaynig V, Longair M, Pietzsch T, Preibisch S, Rueden C, Saalfeld S, Schmid B, Tinevez J-Y, White DJ, Hartenstein V, Eliceiri K, Tomancak P, Cardona A. 2012. Fiji: an open-source platform for biological-image analysis. Nat Methods 9:676–682. doi:10.1038/nmeth.2019 22743772 PMC3855844

[63] Brown AC, Moore CC, Guler JL. 2020. Cholesterol-dependent enrichment of understudied erythrocytic stages of human Plasmodium parasites. Sci Rep 10:4591. doi:10.1038/s41598-020-61392-6 32165667 PMC7067793

[64] Garten M, Nasamu AS, Niles JC, Zimmerberg J, Goldberg DE, Beck JR. 2018. EXP2 is a nutrient-permeable channel in the vacuolar membrane of Plasmodium and is essential for protein export via PTEX. Nat Microbiol 3:1090–1098. doi:10.1038/s41564-018-0222-7 30150733 PMC6158082

