# Supplementary material for: PEXEL is a proteolytic maturation site for both exported and non-exported Plasmodium proteins
Source: mSphere. 2024 Feb 9;9(2):e00393-23. doi: 10.1128/msphere.00393-23 (PMC10900883; doi:10.1128/msphere.00393-23)
Supplement: Supplemental figures and tables — Figures S1-S5 and Table S1. [file msphere.00393-23-s0001.pdf]

MNILNLFICCLSITLLKFIEWEQYNYDCVVAINKKNSIIKNERILDEYENINNSENEEDEYEDY
LDDKGSNEFEQVNYKYLDYVNFTIVKSQSTYQHAVNELFVFLGSQYSTDEKIVVHVKLIDILSL
LFVHYRDNLSNFEHIINSFQDRNKLMNSVEGEYFREFIDERDNYIFDVKNTYYNSQYVNKEKET
ILNKKKTIYEIFQKNWKTDGYRFYSVKNKKKYYYPRKINLDEPRKRKHKKKKKQKNIKCVNMVC
KPLKIEYKSLNKPVNSPVDDNTDVKTMKGEQHIKGQAEDKNHVVQGIENVQQEKKEIMDRIDIQ
NEIQNQVQNDIENEIEYELKNDDNNNNIEESDVMKSYKNQICNADENRTSNVGYVRENSIGPGI
AYRMRKDFFLKDSSFNVITLINSITSNRDNKVVTKLHEGLTKLGITTIEHLIRYTNILAIFFSY
DIFDELYLQIKLVKEYFGLIKYKPNILDTEENEYVVGKKTFYGKYNMIDDEVFVSPNCLSAYCK
LKSVWMQNRNITVKVEKSTTNSLKIMMLGDIGQGFEEEKNFDVQNIYNFMGFNELKSTVQSMKK
WHLENNADFVINLGDNIPNDGSYNFIGNFQWHRLMRELFVFKKKSEKQVHKDLGTNTLSAESIA
NFYNDKIKEMNEGNYENHINYYNDKNLHKIKAQNENNEKENLNDNSIKMNNAKGTSSKINDLQN
VSNTNPYKIEVVDHTKLVESNTSINEKEEKSISSVEERANLYEDDEENDEEEDSNEFASEAIPF
YSVLGEKDYFYFPSEQIQEHYSKRIPGYFMPNNYYCVNYDFTYNPVKKNVNGDEDDDDDEEEEE
GDSGTDRKVKTQEKFRASFIFIDTWALMVGFPIIRNYRAFREQFNWLSKTLYESAKKSDWIFVV
GHHPLISSGRRSDNYSYEEHSFHDIIRDFLFNYHVDAYFSAHDHLMEYIKFGSVDLFINGSSSR
VLFDNSSMGRGYFGKIIGKLYPLSCYVLKTIHTGLKPKGCNINRYSKWYNKSDIGFSTHKLTKD
ELVTQFISSRTGKPLSEKIITKNKKHERKKFYDLDGFAEDRIKELEKKIIDFSVNNPDLINYKI
QEFNENIEKLNLIIKKLKTKEEKEIFKELIQMNNLIFDVSDHLDNVPIEKLKIMSELVSKYNIF
FNKELAGFIVAALERAIQMERKKPHSSDENGSLNDEDKNLIELIESLGYQPEEFLQKYESMTSE
QKVALKNKIGRNISLEDYVNRIKFYVEKKKKEKEKNGNEQEEAEEETEVVEEIDELKEMEKKRK
ESEGDITGEDENEIKEEQKDNEKEDETYEEYLDESQYNDEEIPLVKQVHKDFKKLANQEKKLSE
QKYILLMLASMRKFDIKKYALNLSTKKERIKDVTTSNYLSNIEPRKTFFQLCIELPPDIKRIIN
NFGGVGKRLPFFNFINKLYDEIIKLKDSLNRISR

Figure S1. UIS2 peptides identified by LC/MS-MS from EXP2-BioID2 proximity labeling experiments (1). Peptides are highlighted in red. The PEXEL is underlined and the signal peptide predicted by SignalP 4.1 (2) is shown in bold. The N-terminal most peptide identified corresponds to the processed and N-acetylated PEXEL (see Figure 1B).

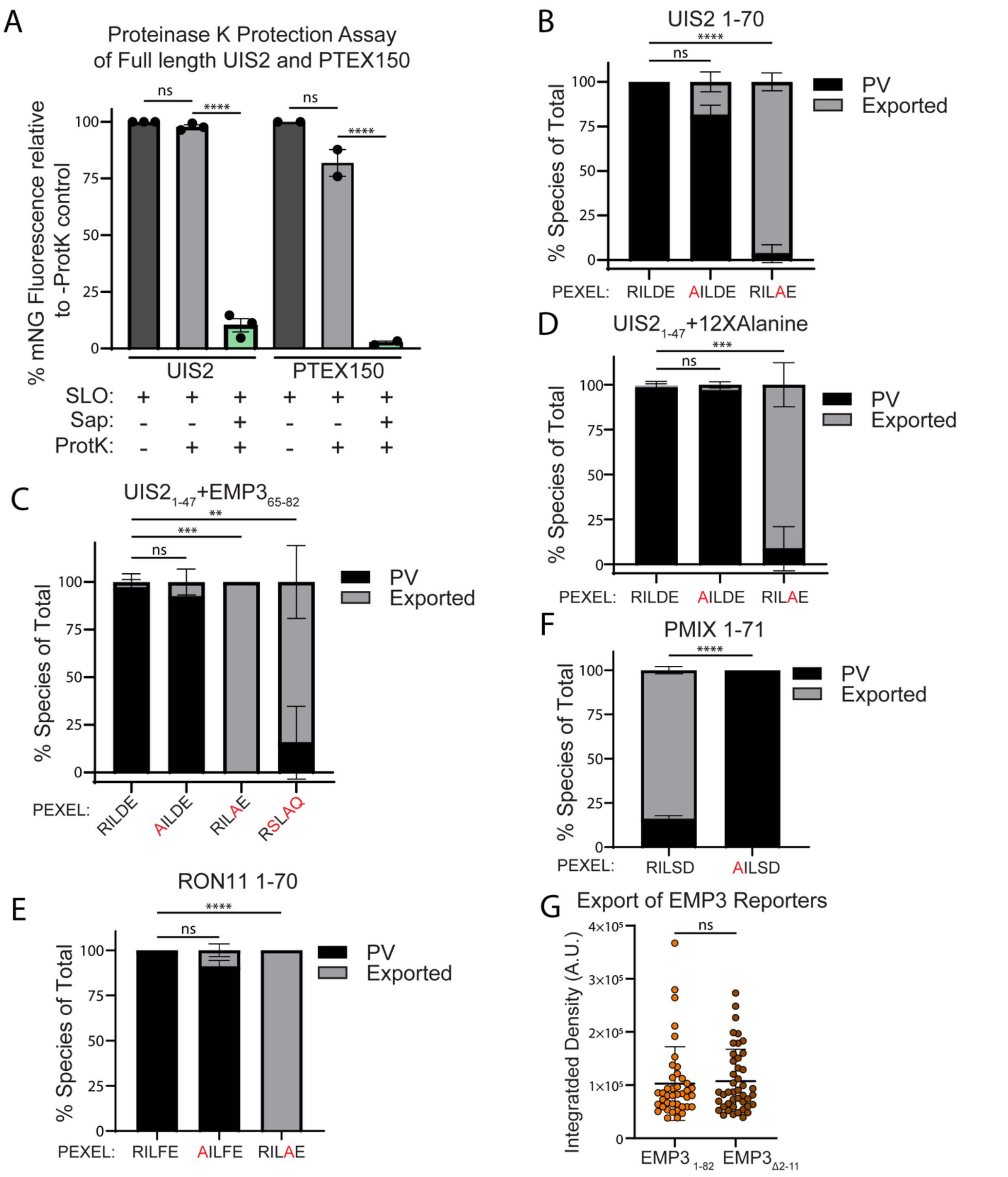

Figure S2. (A) Quantification of protected mNG fluorescence in UIS2-mNG (see Figure 1C) and PTEX150-mNG-kER (3) lines via flow cytometry after treatment with Streptolysin O (SLO), Saponin (Sap), and Proteinase K (ProtK). Mean fluorescent signal was normalized relative to the

+SLO & -Proteinase K treated parasites in each independent experiment. Data are presented as the mean ± SEM from 3 (UIS2) or 2 (PTEX150) independent experiments (ns: not siginficant; ****P<0.0001; unpaired t test). (B-F) Quantification of reporter localization as exported into the RBC or strictly within the PV/parasite from Figures 1-3. Data are grouped by chimera configuration and the PEXEL sequence in each construct is given at bottom with red indicating residue changes from the WT UIS2 (B-D), RON11 (E) or PMIX (F) PEXEL sequence. Statistical analyses were performed by 2-way analysis of variance (ANOVA) using the percent of iRBCs showing export of the WT PEXEL reporter (left most column) in each chimera group as the reference (ns: not siginficant; ***P < 0.01; ****P < 0.001; *****P < 0.0001). G) Quantification of exported mNG in the EMP3 reporters from Figure 4B. Integrated mNG signal density was calculated using Fiji (see methods). Data are presented as means ± standard deviation from 2 biological replicates ($EMP3_{1-82}$ n=41 iRBCs, $EMP3_{\Delta 2-11}$ n=44 iRBCs; ns: not siginficant; unpaired t test).

A

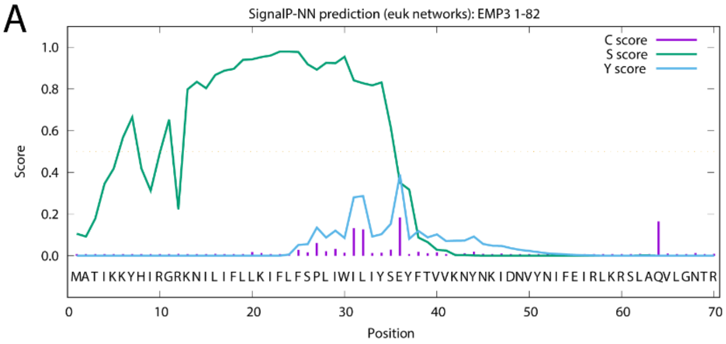

EMP3 1-82

# Measure	Position	Value	Cutoff	signal peptide
max. C	36	0.183	0.32	NO
max. Y	36	0.389	0.33	YES
max. S	23	0.979	0.87	YES
mean S	1 to 35	0.709	0.48	YES
D	1 to 35	0.549	0.43	YES
# Most likely cleavage site between pos. 35 and 36: IYS-EY				

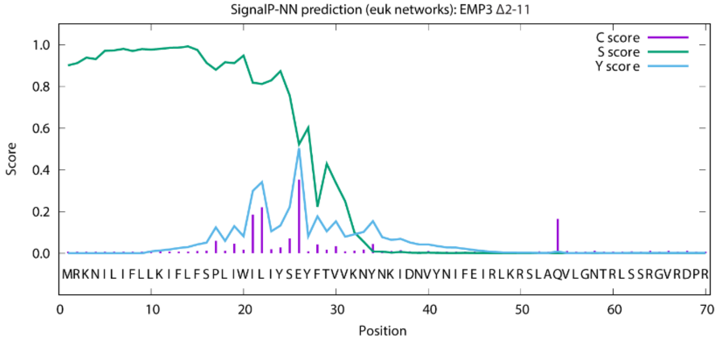

EMP3 Δ2-11

# Measure	Position	Value	Cutoff	signal peptide?
max. C	26	0.353	0.32	YES
max. Y	26	0.504	0.33	YES
max. S	14	0.992	0.87	YES
mean S	1 to 25	0.924	0.48	YES
D	1 to 25	0.714	0.43	YES
# Most likely cleavage site between pos. 25 and 26: IYS-EY				

B

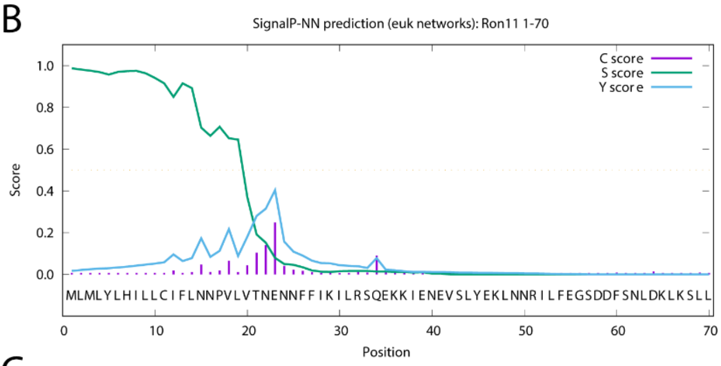

RON11 1-70

# Measure	Position	Value	Cutoff	signal peptide?
max. C	23	0.249	0.32	NO
max. Y	23	0.404	0.33	YES
max. S	1	0.987	0.87	YES
mean S	1 to 23	0.789	0.48	YES
D	1 to 23	0.596	0.43	YES
# Most likely cleavage site between pos. 22 and 23:VTN-EN				

C

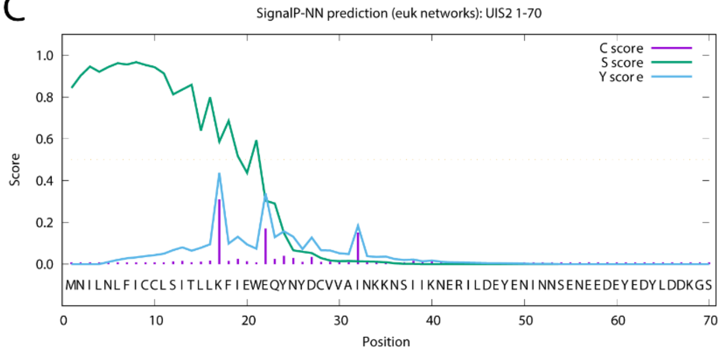

UIS2 1-70

# Measure	Position	Value	Cutoff	signal peptide?
max. C	17	0.31	0.32	NO
max. Y	17	0.437	0.33	YES
max. S	8	0.967	0.87	YES
mean S	1 to 16	0.887	0.48	YES
D	1 to 16	0.662	0.43	YES
# Most likely cleavage site between pos. 16 and 17:TLL-KF				

Figure S3. SignalP3.0 prediction of signal peptide using neural networks (NN) in (A) the reporter that has the first 82 residues of EMP3 fused to mNG or the same reporter lacking residues 2-11 of EMP3, (B) the reporter that has residues 1-70 of RON11 fused to mNG, and (C) the reporter that has residues 1-70 of UIS2 fused to mNG.

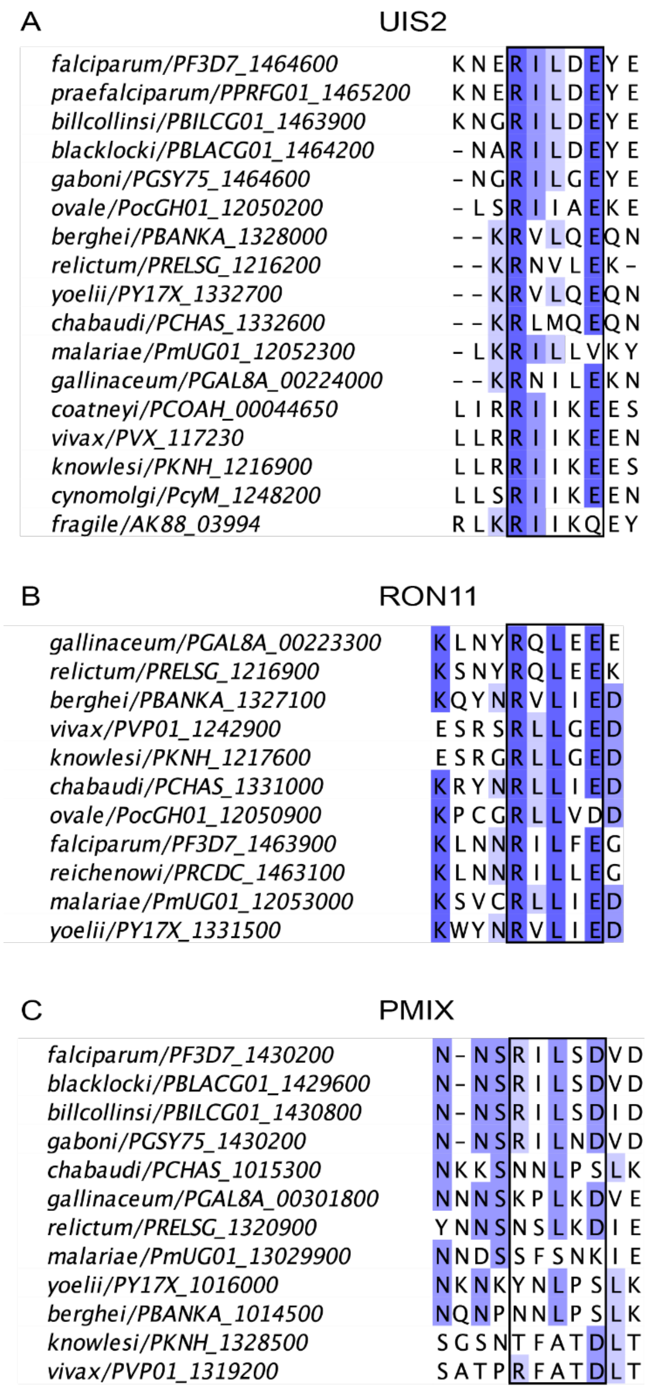

Figure S4. Alignment of (A) UIS2, (B) RON11 or (C) PMIX ortholog sequences from indicated *Plasmodium* spp (species/gene number). Alignments were generated using Clustal Omega (4) and a portion of the alignment corresponding to a 10 amino acid window containing the PEXEL

motif from each *P. falciparum* ortholog was then displayed using Jalview (5). A) Canonical PEXEL sequences are present in UIS2 from *Laverania* species, *P. berghei* and *P. yoelii* while most other species encode a non-canonical PEXEL (RxIxE/D/Q) known to be processed in some contexts (6). P1' residues across all canonical and non-canonical variants are expected to be non-permissive for export (D, Q and K) except for *P. gaboni* (RILGE), *P. ovale* (RIIAE) and *P. gallinaceum* (RNILE). B) Canonical PEXEL sequences are broadly conserved in RON11 although some encode P1' residues expected to be permissive to export. C) Canonical PEXEL sequences are not present in PMIX orthologs outside of *Laverania* species. A non-canonical PEXEL (KxLxE/Q/D) is present in *P. gallinaceum* (KPLKD).

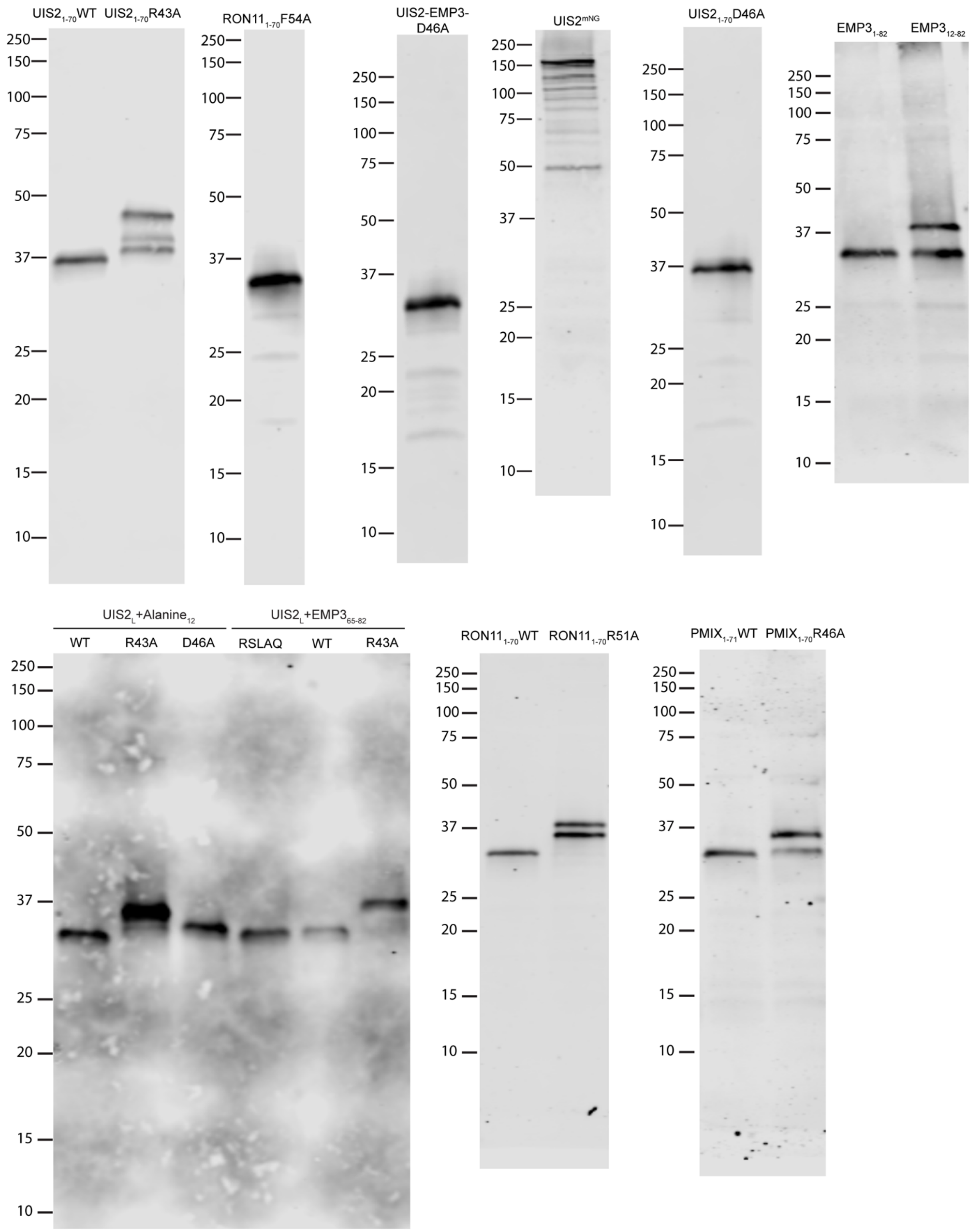

Figure S5. Uncropped western blots from this study.

References

1. Nessel T, Beck JM, Rayatpisheh S, Jami-Alahmadi Y, Wohlschlegel JA, Goldberg DE, Beck JR. 2020. EXP1 is required for organisation of EXP2 in the intraerythrocytic malaria parasite vacuole. Cell Microbiol 22:e13168.
2. Petersen TN, Brunak S, von Heijne G, Nielsen H. 2011. SignalP 4.0: discriminating signal peptides from transmembrane regions. Nat Methods 8:785-6.
3. Fierro MA, Hussain T, Campin LJ, Beck JR. 2023. Knock-sideways by inducible ER retrieval enables a unique approach for studying Plasmodium-secreted proteins. Proc Natl Acad Sci U S A 120:e2308676120.
4. Sievers F, Wilm A, Dineen D, Gibson TJ, Karplus K, Li W, Lopez R, McWilliam H, Remmert M, Soding J, Thompson JD, Higgins DG. 2011. Fast, scalable generation of high-quality protein multiple sequence alignments using Clustal Omega. Mol Syst Biol 7:539.
5. Waterhouse AM, Procter JB, Martin DM, Clamp M, Barton GJ. 2009. Jalview Version 2--a multiple sequence alignment editor and analysis workbench. Bioinformatics 25:1189-91.
6. Schulze J, Kwiatkowski M, Borner J, Schluter H, Bruchhaus I, Burmester T, Spielmann T, Pick C. 2015. The Plasmodium falciparum exportome contains non-canonical PEXEL/HT proteins. Mol Microbiol 97:301-14.

Table S1. Sequences of primers used in this study.

Name	Sequence
P1	AATTTCTACTAAGTGTAGATAAGCTTGCTACTTAAGTTTTTTATTATTTCCTATA
P2	AATTTCTACTAAGTGTAGATTATTACCTTGATATTCTATTAAGGTTTTTTATTATTTCCTATAA
P3	CTATTTTAATTTTTATAATTAATGCTACTCATTAGTGTGTTTATTTACATTTGACCTTAAGATGAGGATAAGAATCTCATAGAATTAATTGAATCTTTGG
P4	TTGCTCACTCCTCCACTTCCCCTTGATATTCTATTAAGGGAATCTTTTAATTTTATTATTTCATC
P5	TTTAGGTGACACTATAGAACTCGAGCTATTGTGATTATTATTTTATTTTTAGTTTTAATTTTTTATGTTTTAACATTTTTTGATTCTAATG
P6	CCAAAGATTCAATTAATTCTATGAGATTCTTATCCTCATCTTAAGGTCAAATGTAAATAAACACACTAATGAGTAGCATTAATTATAAAAATTAAAATAG
P7	ACCTAATAGAAATATATCACCTAGGATGAATATATTAAATCTTTTTATATGTTGTCTTTCTATTACCCTG
P8	CCTCCTCGCCCTTGCTCACGCTAGCGGACCCTTTATCATCTAAATAATCTTCATATTCATCTTC
P9	GTGTTGTAGCAATAAATAAGAAGAATAGTATTATAAAGAATGAAGCAATTCTAGATGAATATGAAAATATTAATAATTCCGAAAATGAAGAAG
P10	CGACTGCTTAACCTTGTATTCCCCAAAACTTCATCTAGAATTCTTTCATTCTTTATAATACTATTCTTCTTATTTATTGCTACAACAC
P11	CGACTGCTTAACCTTGTATTCCCCAAAACTTCATCTAGAATtgcTTCATTCTTTATAATACTATTCTTCTTATTTATTGCTACAACAC
P12	CGACTGCTTAACCTTGTATTCCCCAAAACCTGGGCTAATGATCTTTCATTCTTTATAATACTATTCTTCTTATTTATTGCTACAACAC
P13	ctcgcccttgctcacgctagcCTTAGTTCTAGGATCCCTTACTCCACGACTGCTTAACCTTGTATTCCCCAAAAC
P14	ccttgctcacgctagcAGCTGCGGCTGCAGCTGCAGCGGCTGCAGCGGCTTCATCTAGAATTCTTTCATTCTTTATAATACTATTCTTCTTATTTATTGC
P15	ccttgctcacgctagcAGCTGCGGCTGCAGCTGCAGCGGCTGCAGCGGCTTCATCTAGAATtgcTTCATTCTTTATAATACTATTCTTCTTATTTATTGC
P16	ccttgctcacgctagcAGCTGCGGCTGCAGCTGCAGCGGCTGCAGCGGCTTCtgcTAGAATTCTTTCATTCTTTATAATACTATTCTTCTTATTTATTGC
P17	ACCTAATAGAAATATATCACCTAGGATGGCAACAATAAAAAAATACCATATAAGAGGAAGGAAAAATATTTTAATTTTTTTACTTAAAATATTTTTG
P18	CAATTTTATTATAATTCTTAACAACCGTAAaaTATTCAGAATAAATTAGTATCCATATTAAAGGAGAAAACAAAAATATTTTAAGTAAAAAAATTAAAAT
P19	TGTTAAGAATTATAATAAAATTGACAATGTGTATAATATATTTGAAATAAGACTTAAAAGATCATTAGCCCAGGTTTTGGGGAATACAAGGTTAAGCAGT
P20	ATTATATAACTCGACCTTAAGTCAGGCATAATCTGGAACATCGTAAGGATACGCATA
P21	ACCTAATAGAAATATATCACCTAGGATGAGGAAAAATATTTTAATTTTTTTACTTAAAATATTTTTGTTTTCTCCTTTAATATGGATACTAATTTATTCT
P22	ACCTAATAGAAATATATCAcctaggATGCTGATGTTGTATCTTCATATTTTGTTATGTATTTTCTTAAATAATCCTGTACTTGTTAC
P23	CTCGCCCTTGCTCACgctagcTATCTTTTTTTGAAATGATAATAAACTTTTCAACTTATCTAAGTTTGAG
P24	AGAAATCATCACTACCTTCGAACAAAATAgcATTATTCAACTTTTCATAAAGCGACACCT
P25	AGAAATCATCACTACCTTCtgcCAAAATACGATTATTCAACTTTTCATAAAGCGACACCT
P26	ACCTAATAGAAATATATCAcctaggATGTTTTTTATAAATTTTAAGAAAATAAAAAAGAAACAATTTCCGATATATTTAACTCAACATAG
P27	ctcgcccttgctcacgctagcCGAACACTTATGACATACGTTACATTTTGGTATATTATAATACAATCCT
P28	CTTTTAAGTTTATAAAATAAATAAAAATAAGAAACACTGTTATAATTCTATGTTGAGTTAAATATATCGGAAATTGTTTC
P29	GTGTTTCTTATTTTTATTTATTTTATAAACTTAAAAGATTGTTTCCATATAAATAATTCGgcTATATTAAGTGATGTAGATAAACATAGAGG
P30	tcgcccttgctcacgctagcCGAACACTTATGACATACGTTACATTTTGGTATATTATAATACAATCCTCTATGTTTATCTACATCACTTAATATAgcCG
P31	CGGAATTATTAATATTTTCATATTCtgcTAGAATTCTTTCATTCTTTATAATACTATTCTTCTTATTTATTG
P32	CAATAAATAAGAAGAATAGTATTATAAAGAATGAAAGAATTCTAgcaGAATATGAAAATATTAATAATTCCG
P33	CGACTGCTTAACCTTGTATTCCCCAAAACTTCtgcTAGAATTCTTTCATTCTTTATAATACTATTCTTCTTATTTATTGCTACAACAC
